# Biphasic Granular Bioinks for Biofabrication of High Cell Density Constructs for Dermal Regeneration

**DOI:** 10.1002/adhm.202501430

**Published:** 2025-06-12

**Authors:** Rozalin Shamasha, Sneha Kollenchery Ramanathan, Kristin Oskarsdotter, Fatemeh Rasti Boroojeni, Aleksandra Zielińska, Sajjad Naeimipour, Philip Lifwergren, Nina Reustle, Lauren Roberts, Annika Starkenberg, Gunnar Kratz, Peter Apelgren, Karin Säljö, Jonathan Rakar, Lars Kölby, Daniel Aili, Johan Junker

**Affiliations:** ^1^ Experimental Plastic Surgery Department of Biomedical and Clinical Sciences Linköping University Hospital Linköping 581 83 Sweden; ^2^ Center for Disaster Medicine and Traumatology Department of Biomedical and Clinical Sciences Linköping University Hospital Linköping 581 83 Sweden; ^3^ Laboratory of Molecular Materials Division of Biophysics and Bioengineering Department of Physics, Chemistry and Biology Linköping University Linköping 581 83 Sweden; ^4^ Department of Plastic Surgery Sahlgrenska Academy University of Gothenburg Gothenburg 405 30 Sweden; ^5^ Region Östergötland, Anaesthetics, Operations and Specialty Surgery Center Department of Hand and Plastic Surgery Linköping University Hospital Linköping 581 83 Sweden

**Keywords:** 3D bioprinting, granular bioink, hyaluronan, porous gelatin microcarriers, wound healing

## Abstract

Chronic wounds and severe skin injuries pose significant clinical challenges, as existing treatments like cultured epidermal autografts and tissue engineering strategies fail to regenerate functional dermal tissue effectively. These methods often result in scarring due to poor tissue integration, low cell density, and limited extracellular matrix (ECM) production. Conventional skin tissue engineering relies on time‐intensive cell expansion, producing constructs that lack the complexity of native dermal structures. Here, a bioprintable biphasic granular hydrogel bioink (µInk) based on cell‐laden porous gelatin microcarriers (PGMs) is presented, enabling fabrication of ultra‐high cell density constructs that promote ECM production for dermal regeneration in vitro and in vivo. Primary human dermal fibroblasts are cultured and expanded on PGMs in a bioreactor prior µInk formulation. The cell‐laden PGMs are cross‐linked via copper‐free click chemistry, creating a shear‐thinning granular bioink. The µInk is 3D bioprinted into structurally stable constructs with high cell viability. In vivo, the bioprinted constructs supported neovascularization, hydrogel remodeling, and tissue integration over 28 days. Cells maintained their tissue‐specific phenotype, proliferated, and produced dermal ECM post‐transplantation. The µInk offers a promising approach to generating high cell‐density constructs for scar‐free wound healing and for advancing complex tissue reconstruction.

## Graphical abstract

This study introduces a biphasic granular bioink (µInk) that enables 3D bioprinting of high cell density constructs for dermal regeneration. The bioink promotes cell proliferation, ECM deposition, and neovascularization, leading to tissue integration and reduced scar formation in vivo. This novel approach offers a promising strategy for advancing scar‐free wound healing and tissue engineering.

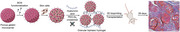

## 1 Introduction

Large wounds heal through the process of granulation tissue formation, yielding a scar that lacks many of the functions as well as the appearance of normal skin, resulting in significant morbidity in affected patients. Burn wounds alone are the fourth most common type of trauma globally with an incidence higher than HIV and TBC combined. According to the World Health Organization, 11 million patients seek hospital attention for burns every year, and 180 000 die.^[^ ^1^ ^]^ Moreover, burns are the fifth most common cause of non‐fatal childhood injuries.^[^ ^2^ ^]^ In a typical hospital setting today, 25% to 40% of beds will be occupied by patients with wounds, and more than half of the health care resources in the outpatient setting are spent on wounds.^[^ ^3^ ^]^ Recent estimates for wound care costs within the US Medicare system amount to USD 96.8 billion annually.^[^ ^4^ ^]^ The skin is also important for our social interactions as well as our body image and self‐awareness.^[^ ^5^ ^]^ Innovations in wound treatment and management hold great promise for improving clinical outcomes and raising quality of life for a large number of patients.

The current gold standard for treating large wounds, such as burns, is split‐thickness skin grafts (STSGs), where the epidermis and a thin part of the dermis is transplanted. The introduction of cultured epidermal autografts (CEAs) by Rheinwald and Green in 1975^[^ ^6^ ^]^ enabled culture expansion of skin, but exclusively targets the epidermal layer. So far, attempts to address healing of the dermis are still no better than using decellularized donor skin.^[^ ^7^ ^]^ Numerous strategies for tissue engineering of skin have been developed,^[^ ^8^ ^]^ but constructs that convincingly recapitulate the qualities of native skin have not yet been demonstrated. In large part, this is due to the limitations of environmental control and a lack of understanding of the regenerative capacities of skin constituents.^[^ ^8^ ^]^ The richness of the dermal extracellular architecture and the complex roles of resident cells are only partially harnessed in canonical approaches to bioengineered skin,^[^ ^8^, ^9^, ^10^, ^11^ ^]^ which largely explains the limited success of many contemporary solutions in clinical applications. Both biologically derived dermal matrices and engineered hydrogels have been used to provide structural support and promote cell attachment, migration, and proliferation, in order to mimic the function of the native extracellular matrix.^[^ ^12^ ^]^ For example, hyaluronan‐, gelatin‐, alginate‐, fibrin‐, and collagen‐based hydrogel scaffolds have been widely reported to recreate a dermal microenvironment that promotes cell adhesion and growth.^[^ ^13^, ^14^, ^15^, ^16^, ^17^ ^]^ The nanoporous polymer network of homogeneous hydrogels can, however, restrain cell migration and proliferation, and the materials must typically undergo degradation to allow for proliferation and cellular infiltration.^[^ ^18^ ^]^ The lack of cell‐to‐cell interactions in low‐cell density constructs can impair cellular crosstalk and formation of native‐like cellular microenvironments, resulting in an inadequate or very slow tissue construct maturation. In addition, balancing degradation rate and overall scaffold integrity to ensure sufficient structural support during tissue maturation requires careful optimization of the materials being used, which can be complicated due to the long culture times required to generate constructs ready for transplantation.^[^ ^19^ ^]^

Granular hydrogels, also known as microgel assemblies, have been proposed as a solution to several of these problems. Granular hydrogels are comprised of micron sized hydrogel particles that are either chemically cross‐linked or physically jammed to form constructs with intrinsic porosity that can facilitate cell infiltration and proliferation. Bottom‐up fabrication of granular hydrogels with tunable microgel size, porosity, composition and different chemical cues could thus enable the preparation of tissue‐mimetic scaffolds with a wide range of properties.^[^ ^20^ ^]^ In addition to their large surface‐to‐volume ratio, the porosity allows for diffusion of nutrients and oxygen into the constructs, promoting long‐term cell viability.^[^ ^21^, ^22^ ^]^ However, physically cross‐linked microgels prepared by jamming, host‐guest complexation or other supramolecular interactions do not typically result in mechanically stable granular hydrogels suitable for transplantation.^[^ ^21^ ^]^ The structural stability of granular hydrogels can be improved by chemical cross‐linking. Caldwell et al.^[^ ^20^ ^]^ utilized click chemistry to obtain cross‐linked polyethylene glycol (PEG) based microgels by alkyne and azide functionalization of the polymer backbone in combination with inverse suspension polymerization and vortexing/sonication. Song et al.^[^ ^23^ ^]^ on the other hand prepared an injectable gelatin‐based jammed microgel that solidified after injection via physical cross‐linking but underwent a secondary chemical cross‐linking process by transglutaminase to create a microgel that was stable in vivo.

The use of 3D bioprinting can further facilitate fabrication of physiologically relevant constructs for transplantation. Bioinks must be shear thinning and undergo rapid cross‐linking post‐printing to ensure structural integrity of printed constructs while maintaining high cell viability. Fang et al^[^ ^25^ ^]^ developed a biphasic microgel bioink for this purpose wherein gelatin methacrylate‐based microgels were infiltrated by a gelatin methacrylate hydrogel precursor that could form a second polymer network after UV/photo cross‐linking to improve post‐printing structural stability. The possibility to trap microgels in a secondary hydrogel network enabled printing of elaborate structures. However, the UV‐mediated cross‐linking process risks damaging delicate primary cells.^[^ ^26^ ^]^ Moreover, biofabrication of dermal constructs requires high cell‐densities. For other tissues, this has previously been accomplished by scaffold‐free bioprinting techniques using spheroid‐based bioinks. While fibroblasts and keratinocytes, both skin‐derived cells, are capable of forming spheroids, the resulting constructs lack the mechanical and structural stability necessary for robust dermal regeneration in clinical applications.^[^ ^27^, ^28^ ^]^ Moreover, fibroblast clustering has been associated with hypertrophic scarring, and in vitro studies have shown that fibroblast spheroids undergo *nemosis*, a process linked to excessive secretion of COX‐2, prostaglandins, and proinflammatory cytokines, which can drive chronic inflammation and impair regenerative healing.^[^ ^29^ ^]^ Alternative strategies that support fibroblast proliferation and ECM deposition without triggering fibrotic pathways are consequently needed.

Here we show a strategy for generating ultra‐high cell density biphasic granular bioinks for dermal regeneration that produce dermal phenotypes and correct ECM composition after transplantation. The ultra‐high cell density constructs were obtained by expanding harvested human primary dermal fibroblasts (HDFs) on porous gelatin microcarriers (PGMs) in a bioreactor prior bioink formulation. The cell‐laden PGMs were then combined with a hyaluronic acid (HA) based hydrogel, cross‐linked with biorthogonal copper‐free click chemistry, resulting in a unique biphasic granular bioink. The PGMs are larger than conventional microgels with an average size of about 250 µm, but the resulting bioink materials showed similar characteristics as granular hydrogels, including shear thinning properties with rapid post‐printing recovery and stability, enabling 3D bioprinting of robust self‐supporting structures and cell‐laden constructs for transplantation.

Fibroblasts are essential for dermal regeneration due to their production of ECM components, such as elastin and collagen, which provide structural support and integrity.^[^ ^30^ ^]^ In wound healing, fibroblasts secrete important growth factors, promoting angiogenesis, epithelialization, and tissue remodeling.^[^ ^31^ ^]^ Additionally, their ability to respond to environmental cues and coordinate the repair process make them crucial for regeneration.^[^ ^32^ ^]^ The culturing of HDFs on the PGMs in bioreactor prior to bioink formulation, enabled rapid expansion of the harvested cells under optimal conditions. By using the HDF‐loaded PGMs as the main component of the granular bioinks, no additional trypsinization of the cells was required and enabled 3D bioprinting of ultra‐high cell density constructs. The HDFs exhibited continuous proliferation and maintained a tissue‐specific phenotype, including the synthesis of key dermal ECM proteins, after bioink formulation and printing. To systematically evaluate these fundamental properties in a controlled setting, printed HDF‐laden constructs were implanted subcutaneously in an immunodeficient animal model. This well‐established approach allowed us to assess ECM deposition, cell viability, vascularization, and tissue remodeling without the confounding variables of an open wound environment, such as inflammation, infection, and contraction. Following implantation, the HDFs remained viable for 28 days, actively contributing to construct remodeling through ECM synthesis and promoting neovascularization and tissue integration. The PGM‐based granular bioinks offers a unique platform for fabricating high‐density cell constructs while providing a cell‐instructive microenvironment that supports tissue formation and integration, ultimately advancing strategies for scar‐free wound healing and dermal regeneration.

## 2 Results and Discussion

### 2.1 Synthesis and Characterization of the Bioinks

**Figure 1:**
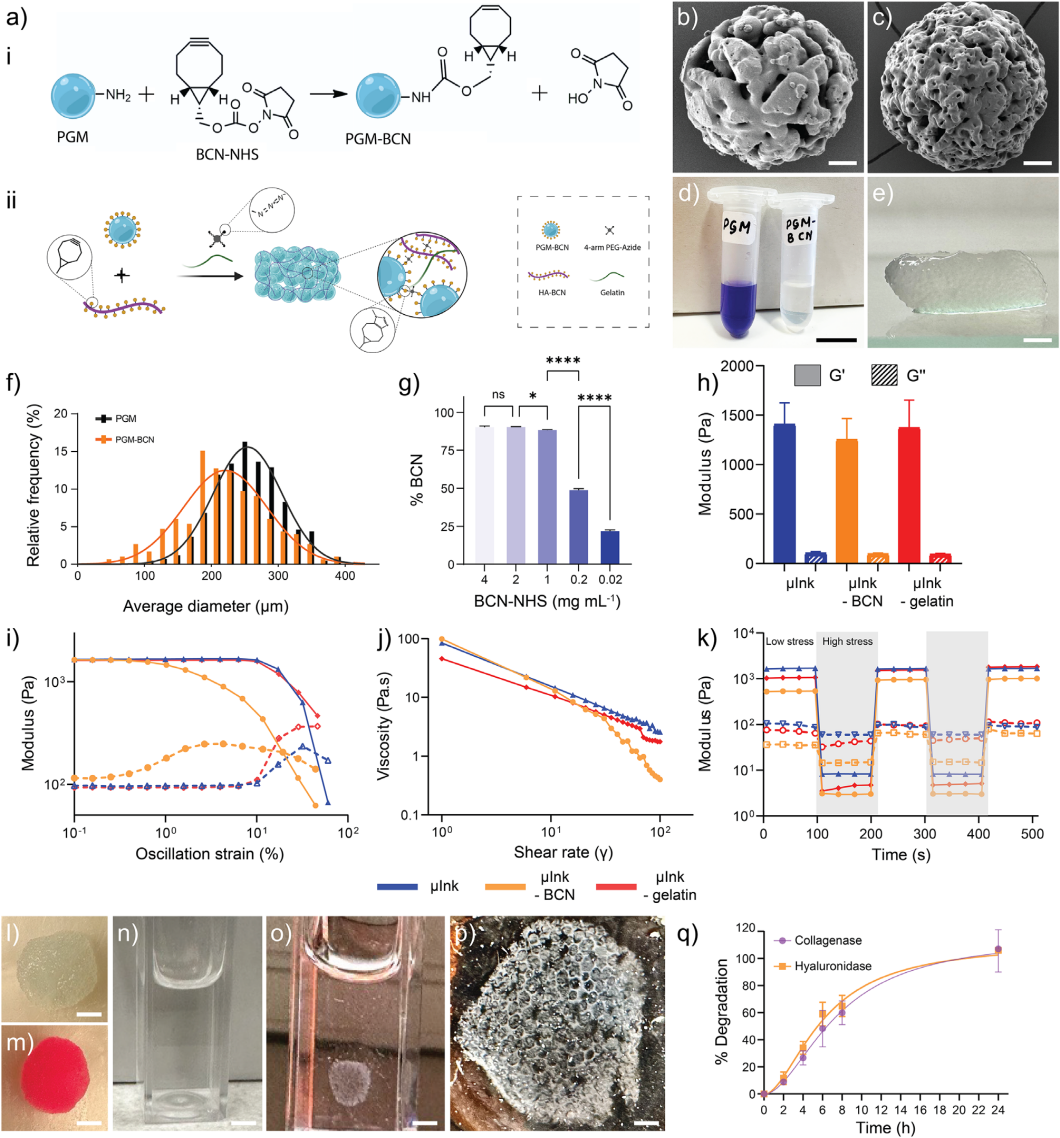
a) (i) Schematic illustration of the conjugation of BCN to PGM. Scanning electron micrographs of b) PGM and c) PGM‐BCN. Scale bars: (b, c) 20 µm. (ii) Schematic representation of the formation of µInk by SPAAC cross‐linking of PGM‐BCN, HA‐BCN, and P(N_3_)_4_. Gelatin was included as a viscosity modifier. d) Ninhydrin test for primary amines in PGM before (left, blue) and after (right, transparent) functionalization with BCN. Scale bar: 1 cm. e) Photograph of the µInk after complete crosslinking at 37 °C. Scale bar: 1 mm. f) histogram of hydrated microcarrier size distribution in µInk. g) % BCN functionalization on PGMs when reacted with different concentration of BCN‐NHS. Rheological properties of the µInk, µInk ‐gel, and µInk ‐BCN; h) Modulus of the granular bioinks at 1 Hz oscillatory frequency after 1.5 h gelation. i) Strain sweeps at 1 Hz. j) Viscosity as a function of shear rate. k) Shear recovery behavior of the granular bioinks. l, m) µInk disc with Cy5‐HA‐BCN before immersion in hyaluronidase solution and µInk disc prepared with Cy3‐PGM‐BCN before immersion in the collagenase solution respectively. Scale bars: 1.5 mm. n) Degradation of the µInk after exposure to hyaluronidase (0.05 mg mL^−1^) after 24 h. Scale bar: 3 mm. o) Selective degradation of PGM‐BCN after exposure of µInk to collagenase (0.05 mg mL^−1^) after 24 h. Scale bar: 2.5 mm. p) Photograph of the remaining microporous HA/PEG hydrogel after degradation of the PGM‐BCN by 0.05 mg mL^−1^ collagenase. Scale bar: 1 mm. q) Degradation profile of µInk prepared with Cy5‐labelled HA‐BCN and Cy3‐labelled PGM‐BCN when exposed to 0.05 mg mL^−1^ hyaluronidase or 0.05 mg mL^−1^ collagenase.

Design of hydrogel bioinks for dermal regeneration requires careful optimization of biofunctionality and mechanical properties to support printing of high cell density constructs that can stimulate formation of new tissues.^[^ ^33^ ^]^ Both hyaluronan (HA) and gelatin alone or in various combinations have been widely used in hydrogels for skin tissue engineering and wound healing applications.^[^ ^34^, ^35^, ^36^ ^]^ We have previously demonstrated possibilities to functionalize HA with bicyclo[6.1.0]nonyne (BCN) to generate HA‐BCN that can be cross‐linked using strain promoted alkyne‐azide cycloaddition (SPAAC) reactions with various azide‐containing cross‐linkers to form biofunctional hydrogels with tunable stiffness.^[^ ^37^, ^46^ ^]^ SPAAC is a biorthogonal reaction that proceeds under physiological conditions without the need for metal catalysts, making it highly suitable for hydrogel encapsulation of sensitive primary cells. Prior studies, including our own,^[^ ^37^, ^38^ ^]^ have consistently shown that SPAAC‐mediated cross‐linking supports high cell viability due to the absence of cytotoxic byproducts and side reactions. Here, to enable fabrication of mechanically robust and bioprintable high cell density hydrogel constructs, we first functionalized porous gelatin microcarrieres (PGMs) with BCN moieties (**Figure** **1**a,i). The PGMs are efficient microporous hydrogel cell‐carrier that are widely used for cell‐expansion.^[^ ^39^, ^40^, ^41^ ^]^ PGMs have also been explored for cell‐transplantation.^[^ ^41^, ^42^, ^43^ ^]^ The BCN‐functionalized PGMs were then used for synthesis of biphasic granular bioink material, here referred to as µInk, employing HA‐BCN and four‐arm PEG‐Az (P(N_3_)_4_) to integrate the PGMs in a hydrogel network (Figure 1a,ii). The PGMs are ≈119±12 µm in diameter when dry (Figure S1c, Supporting Information) and 256±51 µm when swollen in PBS (Figure 1f) and consist of a highly cross‐linked gelatin network derived from collagen type I. They have interconnected pores with a pore size of about 20 µm when hydrated in PBS. Functionalization of PGM with BCN was conducted using carbodiimide chemistry by reacting BCN‐NHS with primary amines in the PGMs.^[^ ^44^ ^]^ A slight decrease in PGM porosity was observed after BCN functionalization, likely due to a reduction in charge of the gelatin which could influence the swelling of the PGM (Figure 1b,c; Figure S1a,b, Supporting Information). The degree of BCN functionalization on PGM‐BCNs was assessed by ninhydrin assay^[^ ^45^ ^]^ which showed a reduction in primary amine content of 91±0.3% after BCN conjugation (Figure 1d) corresponding to 0.54 ± 0.05 nmol BCN per mg of dry PGM‐BCN (Figure S2a,b, Supporting Information). The degree of BCN functionalization could be tuned from 22% to 91% by varying the amount of BCN‐NHS from 0.02 to 4 mg mL^−1^ (Figure 1g; Figure S2c, Supporting Information). To maximize cross‐linking efficiency, we employed the highest achievable degree of BCN functionalization (91%), to ensure robust network formation during bioink gelation and avoid structural heterogeneity or impaired PGM integration. The high degree of functionalization was particularly important given the large size and high surface area of the PGMs, allowing sufficient reactive sites for effective cross‐linking without compromising hydrogel integrity. Addition of four‐arm‐PEG‐azide (P(N_3_)_4_) to PGM‐BCN could in principle result in cross‐linking of the beads but we noticed that the resulting PGM‐gels were very weakly connected and not possible to process further. Thus, we continued to explore the possibilities to integrate the PGM‐BCN into a HA/PEG hydrogel. The conjugation of BCN to HA resulted in a degree of functionalization of 11% as confirmed by ^1^H‐NMR.

By combining PGM‐BCN with a small amount of HA‐BCN and P(N_3_)_4_, a robust biphasic granular hydrogel was obtained (Figure 1e). To further improve the printing properties, we supplemented HA/PEG hydrogels with 1% (w/v) gelatin. The resulting µInk, was comprised of 91.3% of PGM‐BCN, 4% HA‐BCN, 4% P(N_3_)_4_, and 0.7% gelatin by dry weight. The small amount of HA/PEG and gelatin resulted in a biphasic granular hydrogel with high structural stability and interesting viscoelastic properties.

Oscillatory rheology was used to assess the mechanical properties of the hydrogels. Amplitude sweeps (Figure 1i) showed that the µInk behaved as solid‐like elastic hydrogel (G’ > G’’) with a frequency independent storage modulus (G´) of around 1400 Pa (Figure 1h; Figure S3b, Supporting Information), which is on par with the modulus of the reticular dermis.^[^ ^48^ ^]^ Granular hydrogels prepared using native PGM lacking BCN (µInk ‐BCN) were softer (G´ ≈1250 Pa) and showed a lower yield strain (≈19.5%) compared to the µInk (≈45%), demonstrating that the BCN‐functionalization of the PGM contributed to formation of an integrated biphasic hydrogel where hydrated PGMs were connected by the HA/PEG hydrogel. Although the addition of gelatin did not considerably alter the modulus of the µInk, it increased the viscosity of the µInk from 45 to 85 Pa s at γ = 1, which facilitated printing. Interestingly, the µInk was also found to be shear thinning and shear‐recovering (Figure 1j,k). When exposed to a series of alternating low (0.5%) and high strain (500%) cycles, the µInks underwent a reversible transition from a solid‐like elastic state (G’ > G’’) at 0.5% strain to a liquid‐like viscous state (G’’ > G’) at 500% strain. When reducing stress, the µInk regained the original elastic solid‐like behavior even after repeated shear‐recovery cycles. It can be anticipated that the irreversible triazole bonds formed between the BCN and azide moieties rupture under high stress, and when stress is released PGM‐BCN microcarriers within the µInk reorganize and consequently new triazole bonds quickly form between the unreacted BCN and azide moieties causing them to regain its original solid‐like behavior. The shear thinning and shear‐recovery abilities combined with low tan δ value (Figure S3c, Supporting Information) indicates that the µInk indeed is a promising material for 3D bioprinting of structures with high shape fidelity.

To allow for tissue turnover and remodeling during the wound healing process, granular bioink‐based skin constructs should ideally exhibit controlled degradation, thus allowing the cells to replace the µInk with own ECM and integrate with the surrounding tissue. Remodeling of the dermal ECM during wound healing requires concerted interactions of multiple hydrolytic enzymes, most notably by proteases.^[^ ^49^ ^]^ However, hyaluronidase has also been shown to modulate the inflammatory response and contribute to accelerating cutaneous wound healing.^[^ ^50^ ^]^ Here, the specific degradation of the µInk by collagenase (0.05 mg mL^−1^) and hyaluronidase (0.05 mg mL^−1^) was explored using Cy3‐labled PGM‐BCN and Cy5‐labled HA‐BCN. Hyaluronidases catalyze the hydrolysis of 1,4‐b‐d‐glycosidic linkages between N‐acetyl‐galactosamine (or N‐acetyl‐galactosamine sulfate) and glucuronic acid whereas collagenase cleaves peptide bonds in gelatin or collagen. The µInk disc disintegrated completely when exposed to hyaluronidase due to the degradation of the HA/PEG hydrogel dissolving the interconnected microcarrier network (Figure 1n). In contrast, addition of collagenase led to specific degradation of the PGM‐BCN resulting in an intact but microporous HA/PEG hydrogel (Figure 1o,p). The HA‐BCN and PGM‐BCN components were fully degraded after 24 h when treated with hyaluronidase and collagenase, respectively (Figure 1q).

### 2.2 Morphology and Porosity of the µInk

**Figure 2:**
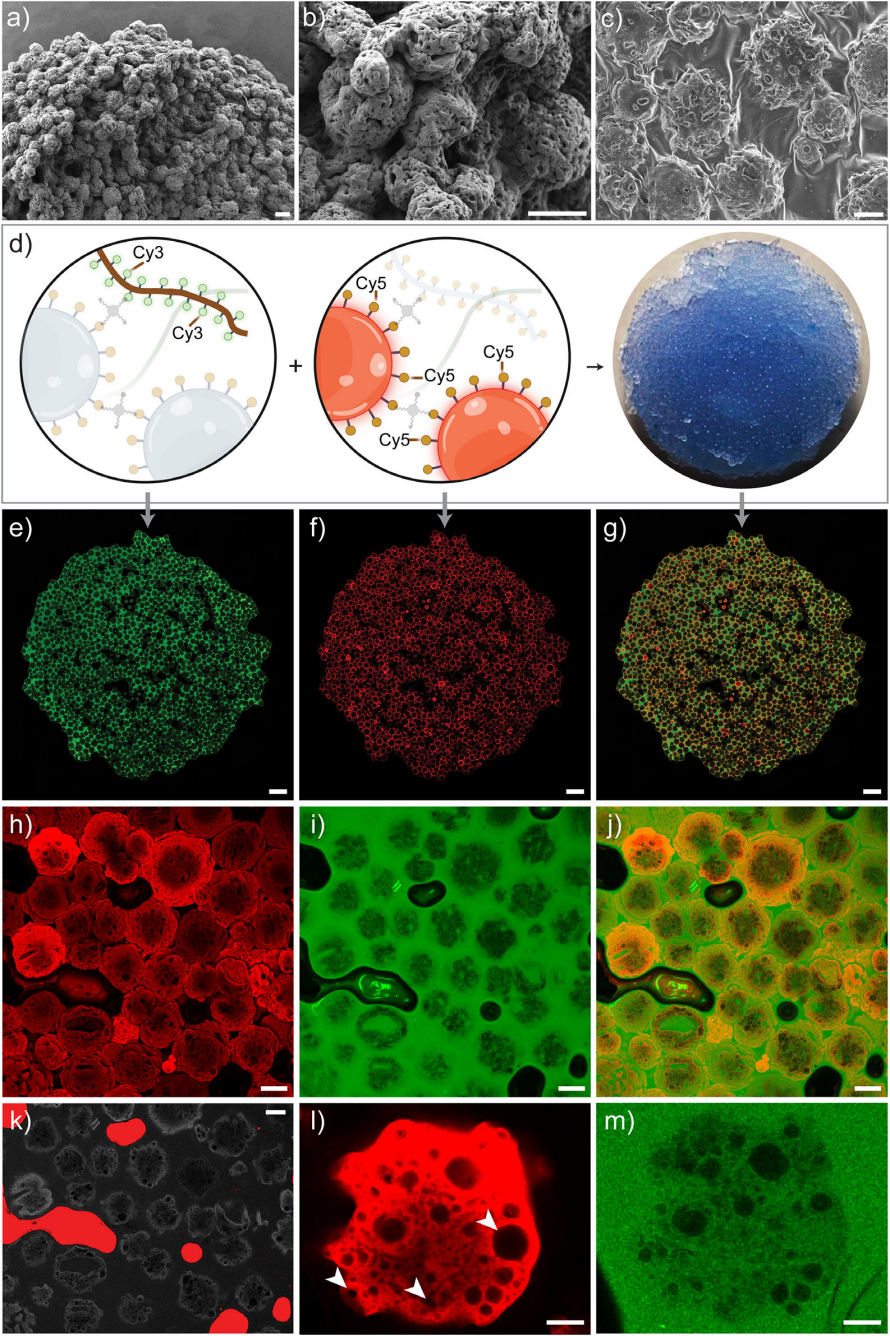
a) – c) SEM images of the µInk. Scale bars: a) 100 µm, b) 50 µm, and c) 25 µm. d)µInk was prepared using Cy3 labelled HA‐BCN and Cy5 labelled PGM‐BCN. Confocal fluorescence stitched tile scan slices of e) Cy3 labelled cross‐linked HA‐BCN, f) Cy5 labelled PGM‐BCN, and g) merged 2D slices. Scale bars (e‐g): 500 µm. Maximum intensity projections of h) Cy3 labelled cross‐linked HA‐BCN, i) Cy5‐labelled PGM‐BCN, and j) composite. k) 2D slice of the µInk highlighting the porosity outside the PGM‐BCN (red) and in the PGM‐BCN after formation of the µInk (black). Scale bars h‐k): 100 µm. Fluorescence confocal image of l) PGM‐BCN in the µInk; white arrows represent the surface pores filled in by the crosslinked HA‐BCN and m) surrounding crosslinked HA‐BCN. Scale bars (l, m): 25 µm.

**Figure 3:**
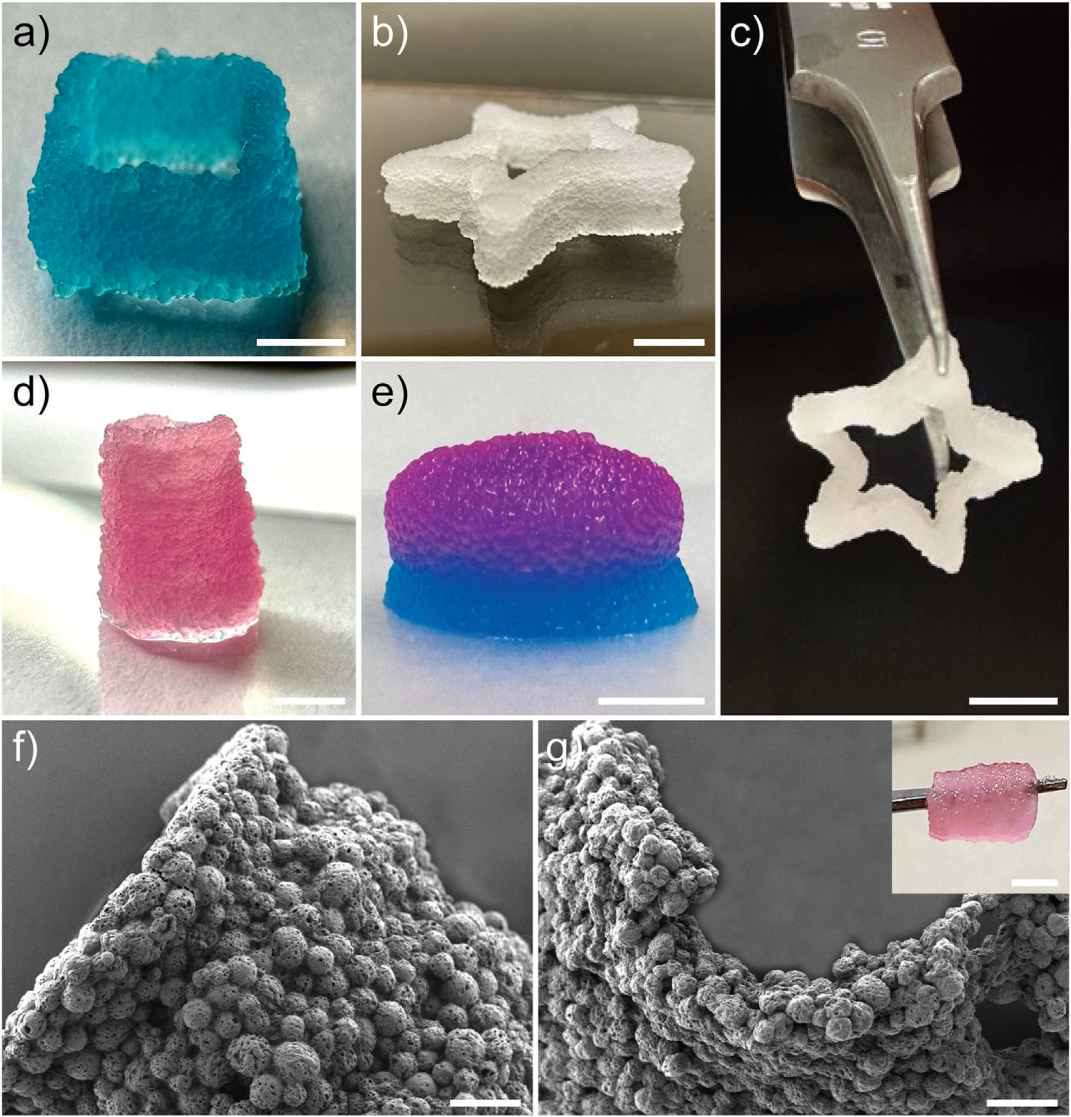
3D printed structures using µInk. a) Hollow square with six layers, b,c) five layered five‐pointed star, d) hollow cylinder comprising ten layers, and e) disc printed with two layers with Cy‐5 labelled PGM‐BCN (bottom) and two layers with Cy‐3 labelled PGM‐BCN (top). f) SEM image of the printed hollow cylinder shown in (d). g) SEM image of the printed cylinder shown in (d) after 45 days of incubation in HDFM. Inset shows photograph of the printed hollow cylinder after 45 days of incubation in PBS. Scale bars: (a‐e) 2.5 mm and (f, g) 200 µm.

SEM images of the µInk (**Figure** **2**a–c) clearly show that the PGMs were homogenously integrated in the cross‐linked HA/PEG hydrogel. The quantity of the HA/PEG hydrogel was optimized to effectively form and keep the shape fidelity of the µInk by forming a secondary continuous polymeric network. Fluorescence confocal 2D tiled scans (Figure 2e–g) and 3D images (Figure 2h–j) demonstrate that the PGMs occupied the major volume of the µInk, which is expected as the PGMs comprise > 91% of the hydrogel by weight. In addition to the inherent porosity of the microspheres, jamming of the PGMs in the µInk resulted in additional pores in between the beads corresponding to a porosity of 6 ± 2%, Figure 2k; Figure S4g,h, Supporting Information). Fluorescence confocal images of a single microsphere and the HA/PEG hydrogel surrounding it in the µInk (Figure 2l,m) suggest that HA/PEG hydrogels also occupy some of the pores of the PGMs close to the surface of the microsphere (white arrows) while leaving most of the inner pores empty. The µInk can thus retain high porosity, which can further facilitate cell proliferation, infiltration and spreading.^[^ ^51^ ^]^

### 2.3 Exploring µInk Printability

The possibility of using the µInk as a 3D bioink material was first optimized and confirmed by extrusion through a syringe using a 20G cone (Videos S1 and S2, Supporting Information). The extrusion and integrity of the resulting structures were greatly improved by incubating the µInk at 37 °C for 75 min prior to extrusion to initiate a partial cross‐linking of the granular hydrogel. Extruding the µInk into 3D printed molds resulted in structures with excellent shape stability after an additional incubation at 37 °C for 60 min to fully cross‐link the materials (Figure S5a, Supporting Information). The printed hydrogels were mechanically robust and could be easily handled using forceps (Figure S5b, Supporting Information; Video S3, Supporting Information). Unlike jammed microgels, the µInk constructs did not require any post‐treatments, such as s secondary cross‐linking step^[^ ^23^, ^52^, ^53^ ^]^ to stabilize the structures after 3D printing. After confirming that the µInks could indeed be extruded and generate stable structures, we continued to explore the possibilities generating more complex architecture using a 3D bioprinter. Note that the printing resolution was limited by the relatively large size of the PGMs, however, the printing resolution was more than sufficient for printing of dermal constructs. To prevent clogging, a standard conical bioprinting nozzle with a nozzle diameter of 580 µm (20G) was used, which is about twice the average diameter of the PGMs. Due to the careful optimization of the rheological properties of the µInks, robust free‐standing multilayer structures (> 10 layers) could be printed without the use of any support bath (**Figure** **3**a–e; Figure S6 and Video S4, Supporting Information). SEM images of a printed µInk cylinder (Figure 3f) show a continuous structure without any discernable boundaries between the printed layers. The microarchitecture and structural stability of the printed structures were preserved even after incubation in cell culture medium (HDFM) for more than 45 days at room temperature (Figure 3g), clearly indicating the possibilities of printing constructs for in vivo transplantation.

### 2.4 Cell Seeding on PGM‐BCN

**Figure 4:**
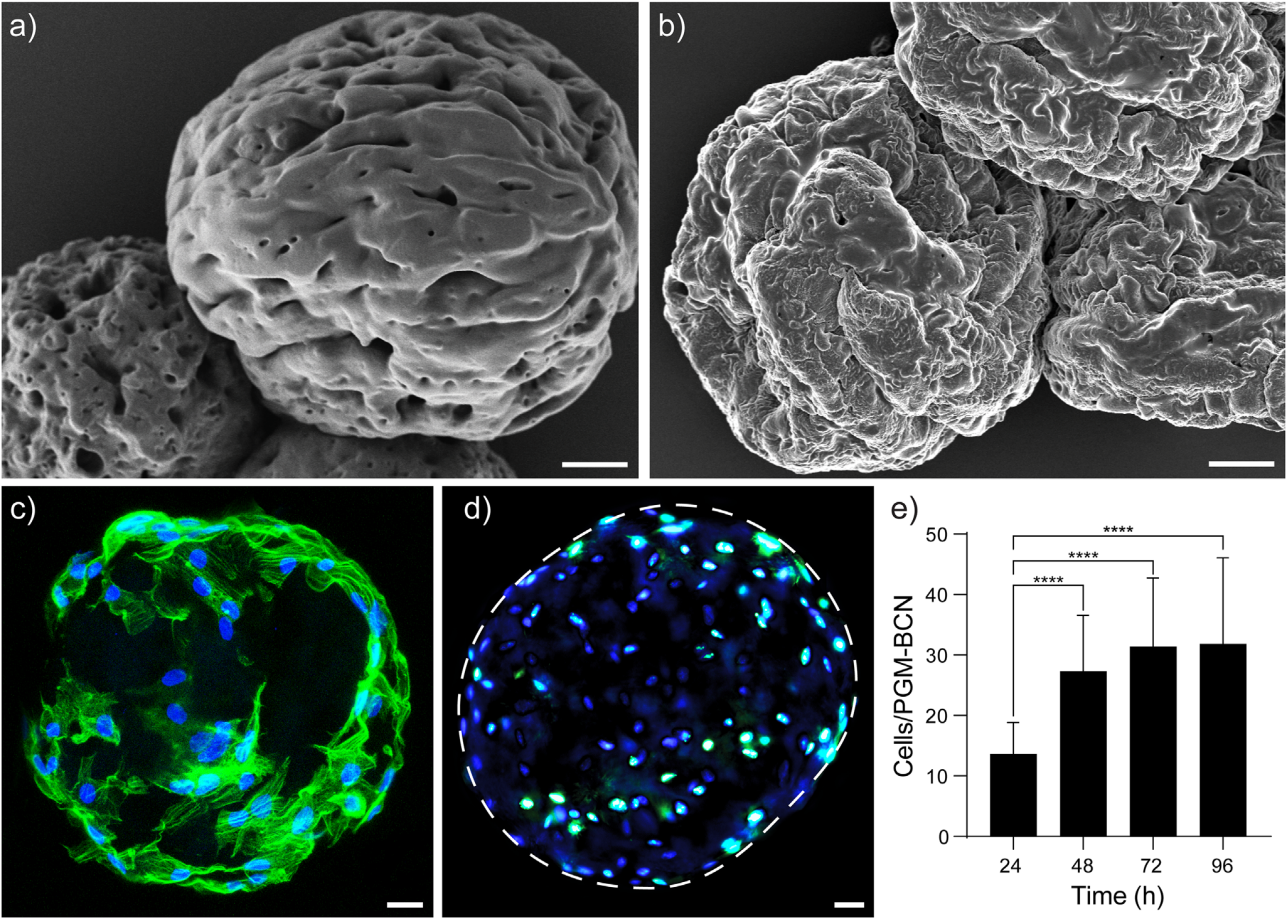
a) SEM image of PGM‐BCN. b) SEM image of cell‐laden PGM‐BCN. c) PGM‐BCN seeded with HDFs stained with phalloidin (green) and DAPI (blue). d) PGM‐BCN (circled in white) seeded with HDFs stained with antibodies for Ki67 (green) and DAPI (blue) and e) nuclei per PGM‐BCN over time, analyzed using one‐way ANOVA with Tukey´s HSD post‐hoc test (n = 150). Scale bars: 20 µm (a, b) and 10 µm (c. d). *** = p < 0.005, **** = p < 0.001.

HDFs are essential for dermal regeneration as they produce extracellular matrix components such as collagen, which are essential for structural support. To generate HDF‐laden µInks, HDFs were seeded on PGM‐BCN and cultured for three days in a stirred flask bioreactor. To assess HDF attachment to the PGM‐BCN, samples were visualized using SEM (**Figure** **4**a–b), confirming that the cells were interacting with the surface and spreading on the PGM‐BCN.

Upon further investigation using immunofluorescent staining for Ki67 (Figure 4d, green),^[^ ^54^ ^]^ a subset of cells was demonstrated to be in a proliferative state (G_1_, S, G_2_, or M). Cell proliferation was further evident by quantification of DAPI‐stained nuclei over time (Figure 4e), where the number of cells on the PGM‐BCN was shown to significantly increase from 13.6 ± 5.2 at 24 h to 27.3 ± 9.3 and 31.4 ± 11.4 at 48 h and 72 h respectively. Cells per PGM‐BCN plateaued after 72 h at 31.9 ± 14.2 at 96 h (n = 150 PGM‐BCN).

### 2.5 Protein and Gene Expression of HDF on PGM‐BCN

**Figure 5:**
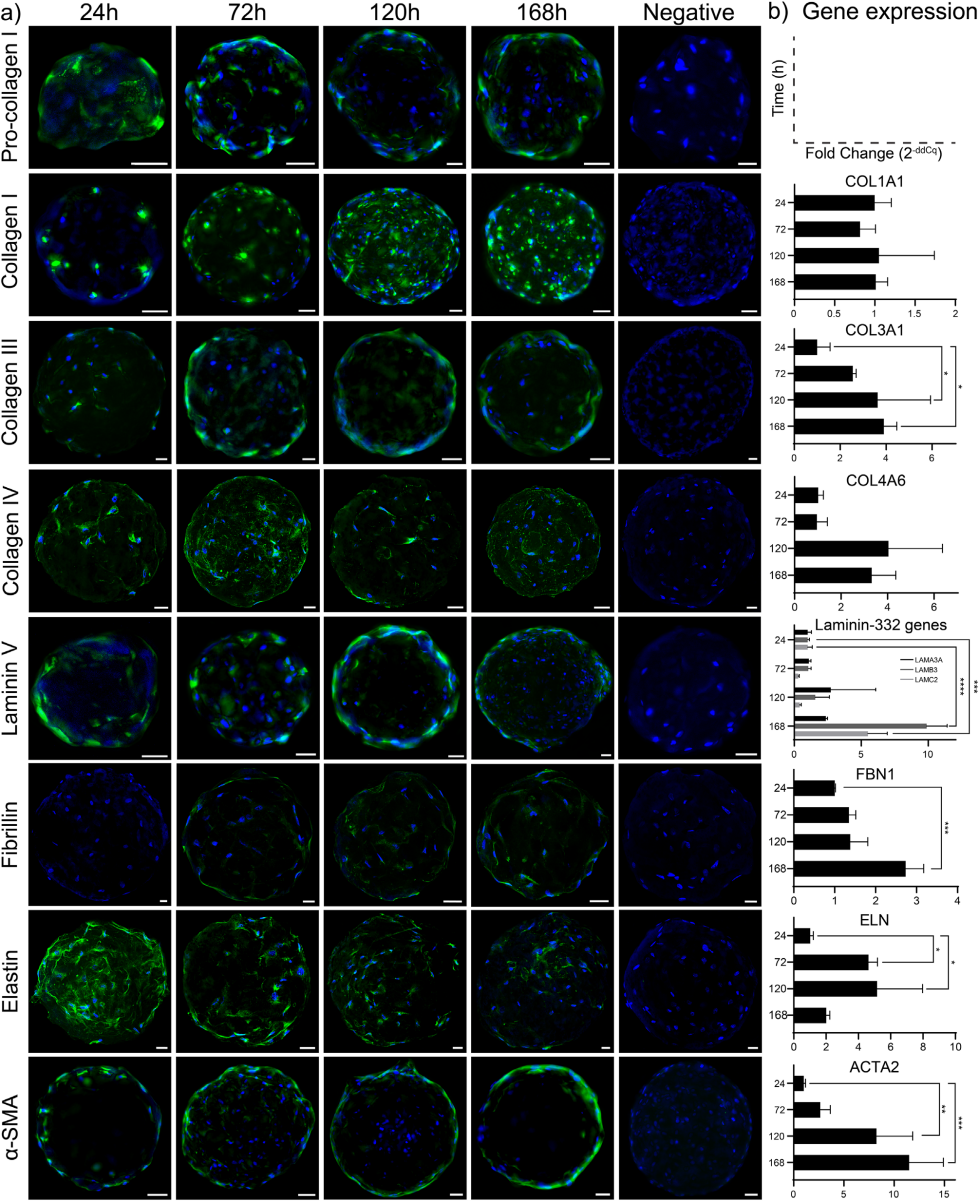
a) Cell‐laden PGM‐BCN stained using antibodies (green) against pro‐collagen I, Collagen I, Collagen III, Collagen IV, Laminin‐332, Fibrillin, Elastin, and alpha‐smooth muscle actin (α‐SMA) after 24, 72, 120, and 168 h of culture. Non‐specific binding was controlled by omission of primary antibodies. Nuclei were stained using DAPI (blue). Scale bars: 25 µm. b) Fold change of corresponding genes compared to endogenous control, analyzed using one‐way ANOVA with Dunnett´s post‐hoc test (n = 3 biological replicates per gene and timepoint). * = p < 0.05, ** = p < 0.01, *** = p < 0.005, **** = p < 0.001.

ECM components play a key role in each stage of wound healing by providing structural support, enabling intercellular interactions, and contributing to the formation of the tissue microenvironment.^[^ ^55^ ^]^ Absence of ECM in a wound may result in insufficient tissue regeneration due the cirtical role of cell‐ECM interactions in tissue formation. Biomatrices may therefore be used in combination with grafting techniques to mimic the role of the ECM at the wound site, resulting in decreased scar formation and wound contraction.^[^ ^56^ ^]^ Fibroblast contractility is promoted by expression of alpha‐smooth muscle actin (α‐SMA), which is a marker for myofibroblast differentiation.^[^ ^57^ ^]^ Myofibroblasts synthesize ECM components of the fibrocontractile matrix present in remodelling tissues, such as collagen types I and III.^[^ ^58^ ^]^ The synthesis of ECM‐components by HDFs cultured on PGM was therefore investigated by immunofluorescent staining as well as qPCR analysis of their respective genes (**Figure** **5**). Pro‐collagen I and collagen I are both present at the first timepoint (24 h). Whereas expression of pro‐collagen I was constant over time, collagen I increased over time. The corresponding gene, COL1A1, was however not showing any increase in gene expression over time, remaining at 1.01 ± 0.20 at 24 h, and 1.01 ± 0.14 at 168 h. This suggests a stable output of collagen I, that accumulates in the pericellular space. Collagen III protein expression showed a steady increase over time, also indicating a gradual accumulation of the protein on the PGMs. This was corroborated by the expression of the COL3A1 gene, exhibiting a steady significant increase from 1.07 ± 0.48 at 24 h to 3.93 ± 0.51 at 168 h, which is a near four‐fold increase over this time course. The ratio between collagen I and III is a qualitative marker for fibrotic ECM, but our analysis is not sufficient to determine their relative structural or stoichiometric distribution in the constructs. Collagen IV was abundantly present at the first timepoint, increasing to a maximum expression at 120 h, while decreasing by the last timepoint. This could be confirmed by the gene expression of COL4A6, which started at 1.02 ± 0.22 at 24 h, increasing to 4.04 ± 2.32 at 120 h, and decreasing to 3.32 ± 1.04 at 168 h. Laminin V was also present at 24 h, and increased significantly over time. Laminin V is expressed by three genes, LAMA3A, LAMB3, and LAMC2. Expression of LAMA3A was steady over time, showing a non‐significant increase from 1.02 ± 0.27 at 24 h to 2.36 ± 0.13 at 168 h. However, both LAMB3 and LAMC2 showed a significant increase in gene expression over time. LAMB3 exhibited a tenfold increase from 1.01 ± 0.13 at 24 h to 9.95 ± 1.47 at 168 h and LAMC2 exhibited a fivefold increase from 1.03 ± 0.28 at 24 h to 5.60 ± 1.39 at 168 h. Fibrillin is not a direct participant in the molecular events of wound healing, however, it plays a role in maintaining proper architecture of the ECM by providing structural support and maintaining tissue elasticity.^[^ ^59^ ^]^ There were no signs of positive fibrillin staining at 24 h, but expression of fibrillin was present at 72 h and remained at a steady level throughout the experiment. Gene expression of FBN1 exhibited a significant three‐fold increase from 1.00 ± 0.02 at 24 h to 2.73 ± 0.44 at 168 h. Elastin provides elasticity in tissues, which is crucial for tissues subject to mechanical stress, such as the skin.^[^ ^60^ ^]^ During granulation tissue maturation, there is an increased deposition and organization of elastin, contributing to restoration of the tissue integrity.^[^ ^60^ ^]^ Here, elastin was expressed at 24 h, and decreased over time. This was confirmed by ELN gene expression, which started at 1.01 ± 0.20 at 24 h, and increased to 4.62 ± 0.55 and 5.16 ± 2.82 at 72 and 120 h, respectively, and then decreased to 2.01 ± 0.22 at 168 h. α‐SMA was expressed at 24 h and increased steadily throughout the experiment. This was confirmed by ACTA2 gene expression, which showed a significant increase from 1.01 ± 0.17 at 24 h, 8.62 ± 3.229 at 120 h and 11.74 ± 2.87 at 168 h. Taken together, the gene and protein expression data of HDFs cultured on PGM‐BCN in vitro suggests the formation of differentiated microtissues with a dermis‐like ECM. The cells produced many of the major ECM components expected of HDFs, and there is evidence of accumulation of de novo synthesized ECM on the constructs. Cells developed a myofibroblast‐like phenotype, with concomitant expression of collagen I and III, and production of fibroelastic components such as fibrillin and elastin. The production of collagen IV and laminins is interesting in this context since it is not obvious whether these components contribute to the formation of a basement membrane in the absence of epithelia.

### 2.6 3D Bioprinting of HDF Laden µInk

**Figure 6:**
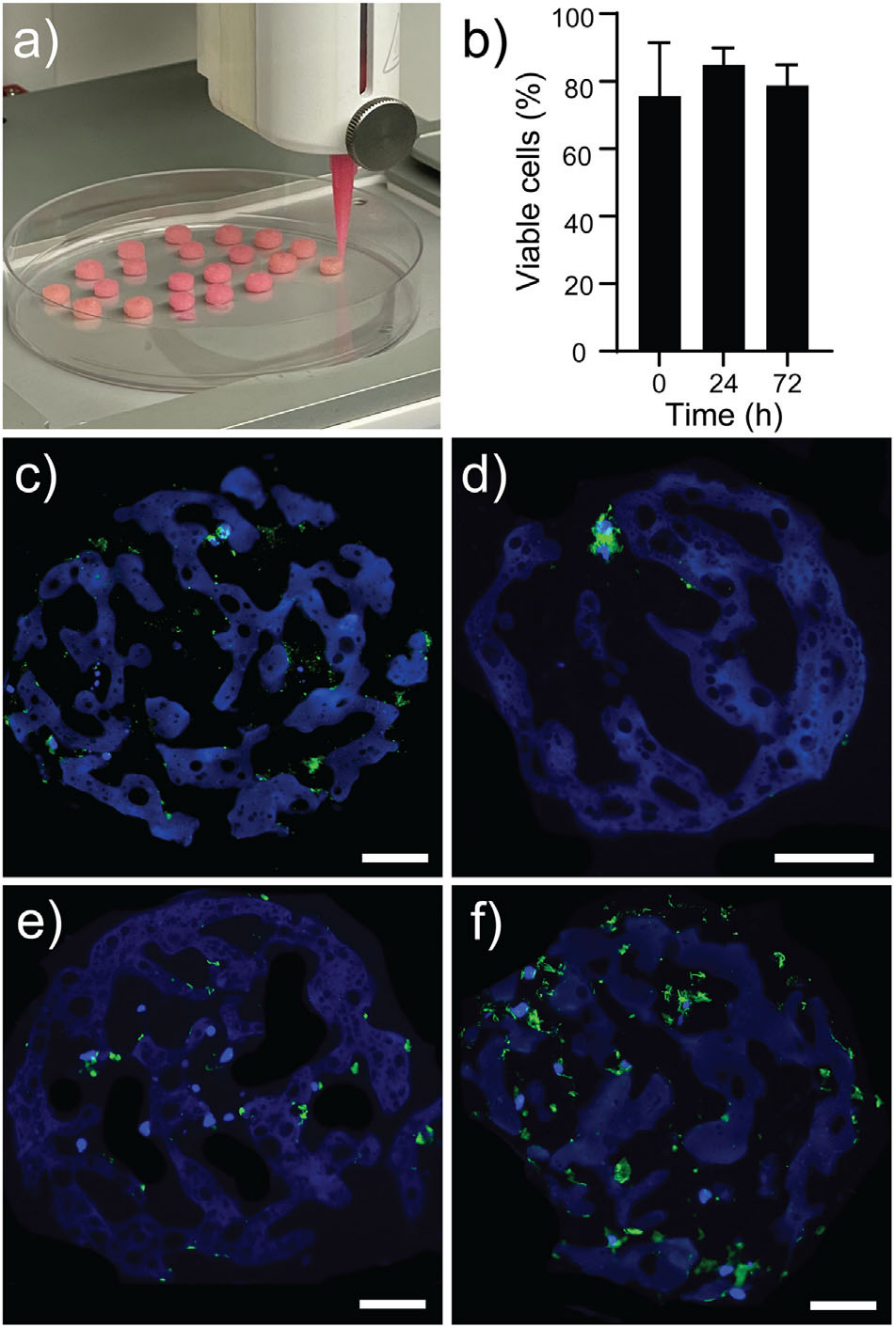
a) Bioprinting of HDF‐laden PGM‐BCN. b) Cell viability expressed as the percentage of live cells relative to the total number of cells within each construct at 0 h, 24 h, and 72 h after printing, analyzed using one‐way ANOVA with Holm‐Šídák test (n = 3‐6). c–f) Representative confocal images of printed constructs stained with phalloidin (green, F‐actin) and DAPI (blue, nuclei) at 24 h (c), 72 h (d), 120 h (e), and 168 h (f). Images show optical sections (6 µm) of the constructs; only a subset of cells is visible. Scale bars: 50 µm.

Despite the high cell density in the PGM‐BCN after 3 days of culture, the HDF‐laden µInk could still form a stable granular bioink that was printable using an extrusion bioprinter. The 3D bioprinted structures retained their shape after cross‐linking (**Figure** **6**a). Cell viability after bioprinting of the constructs was assessed using Zombie Red. Cell viability is presented as the percentage of live cells relative to the total number of cells within each construct at the respective time points and was 78.9 ± 6.0% at 0 h, 84.5 ± 5.0% at 24 h, and 75.6 ± 15.9% at 72 h (Figure 6b). Examples of the images used for viability assessments can be found in Figure S7 (Supporting Information). Staining of F‐actin using phalloidin allows for visualization of the cytoskeleton,^[^ ^61^ ^]^ which can be used to assess changes in cell number over time as well as cell distribution within the bioprinted constructs. Representative images of phalloidin staining within a printed construct (Figure 6c–f, green) show that the phalloidin signal increases over time, indicating that cells continued to proliferate within the constructs. Note that the phalloidin‐stained images represent thin optical sections of the constructs rather than full 3D volumes, and thus only show a fraction of the total cell population. The uncropped images of the phalloidin‐stained constructs are available in Figure S8 (Supporting Information).

### 2.7 Post‐Implantation Macroscopic Assessment

**Figure 7:**
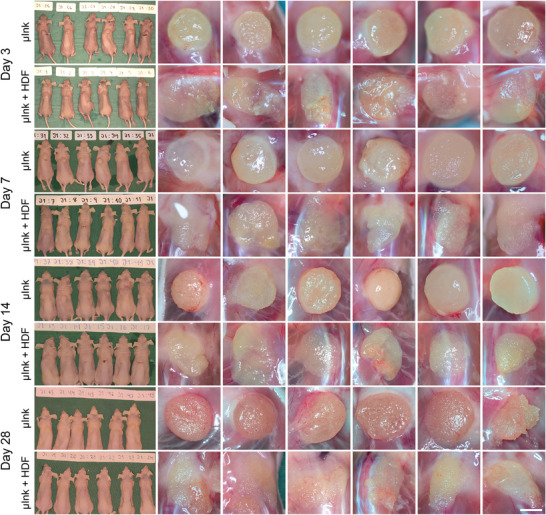
Macroscopic assessment of implanted µInk and µInk + HDF. Scale bar: 3 mm.

To investigate biocompatibility of the µInk as well as cell‐mediated remodeling of the 3D bioprinted constructs in a physiological environment, constructs with and without HDFs were implanted subcutaneously in female Balb/c mice. All animals remained in good health throughout the experiment. Optical assessment of the implanted constructs revealed a higher degree of shape preservation in the cell‐free group compared to the cell‐laden constructs (**Figure** **7**). On the day of implantation, all constructs in both groups had a stable and uniform shape, although cell‐laden constructs appeared more brittle during handling, likely due to cells on the PGMs interfering to some extent with the cross‐linking. On day three post‐implantation, cell‐laden constructs had already undergone significant shape changes and flattened. By contrast, the cell‐free constructs retained their shape during the entire implantation experiment, showing signs of shape change only by day 28. They were still visible through the skin of the mice at days 14–28, whereas cell‐laden constructs were less so. These findings indicate an ongoing remodeling of the cell‐containing constructs mediated by the transplanted cells.

### 2.8 Histological Assessment of Implanted µInk Constructs

**Figure 8:**
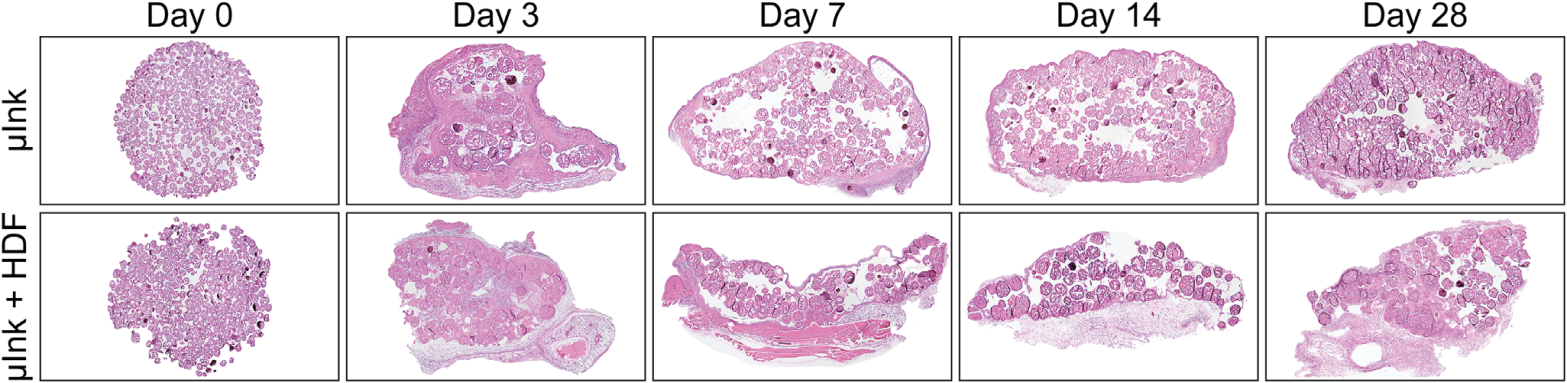
Hematoxylin‐eosin staining of transplanted µInk and µInk + HDF. Samples were taken from the three‐, seven‐, fourteen‐ and twenty‐eight‐day timepoints.

Hematoxylin‐Eosin (HE) staining was used for visualization of cell morphology, tissue architecture, and construct composition.^[^ ^62^ ^]^ The results provided crucial information about the in vivo behavior of the bioprinted constructs, including structural integrity and cellular infiltration. **Figure** **8** shows one representative sectioned sample per group and timepoint. Similar porosities were seen in both the µInk and the µInk + HDF constructs, with similar overall morphology at Day 0 as in the non‐transplanted constructs (Figure 2k; Figure S4g,h, Supporting Information). The µInk + HDF constructs displayed a higher degree of ECM synthesis and tissue remodeling, consistent with the macroscopic observations. No fibrous capsule formation or excessive cell infiltration were noted at any of the timepoints.

**Figure 9:**
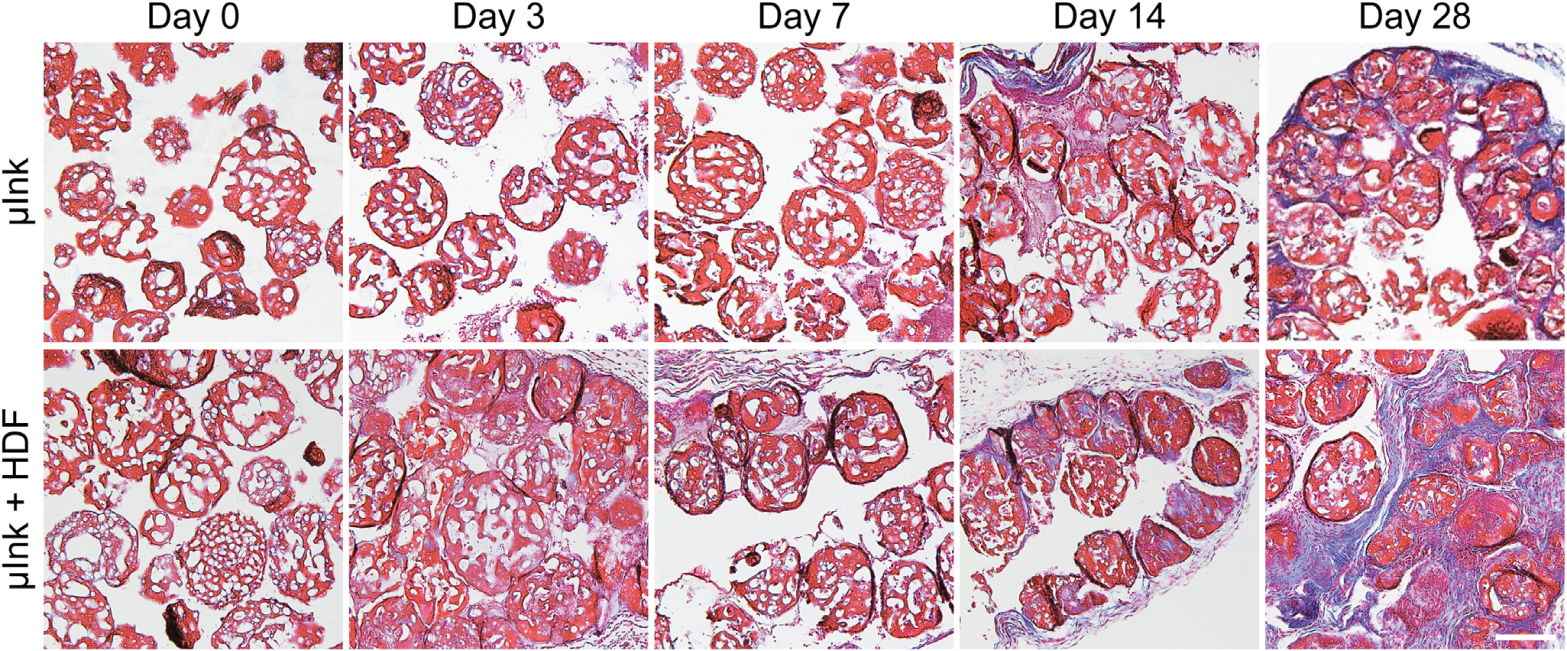
Representative trichrome stained histological sections of implanted µInk and µInk + HDF over time. Trichrome staining highlights collagen fibers in blue/purple and cell nuclei in dark blue. Scale bar: 100 µm.

Trichrome staining was used to provide further insights in the wound healing process by visualizing collagen fibers, distinguishing between newly formed and existing tissue (**Figure** **9**).^[^ ^63^ ^]^ Collagen fibers, visible in blue/purple, were shown to increase over time and were abundantly present at the 28‐day timepoint in the µInk + HDF group. This indicates a more mature ECM production, compared to transplantation of µInk without cells. This formation of a dermis‐like ECM was localized both inside and surrounding the PGMs in the bioprinted constructs. Interestingly, collagenous matrix formation was also observed on the µInk constructs not containing cells, but to a lesser extent. This suggests that the bioink stimulates cell ingrowth from adjacent tissue.

### 2.9 Proliferation of Transplanted Cells and Host Cell Infiltration

**Figure 10:**
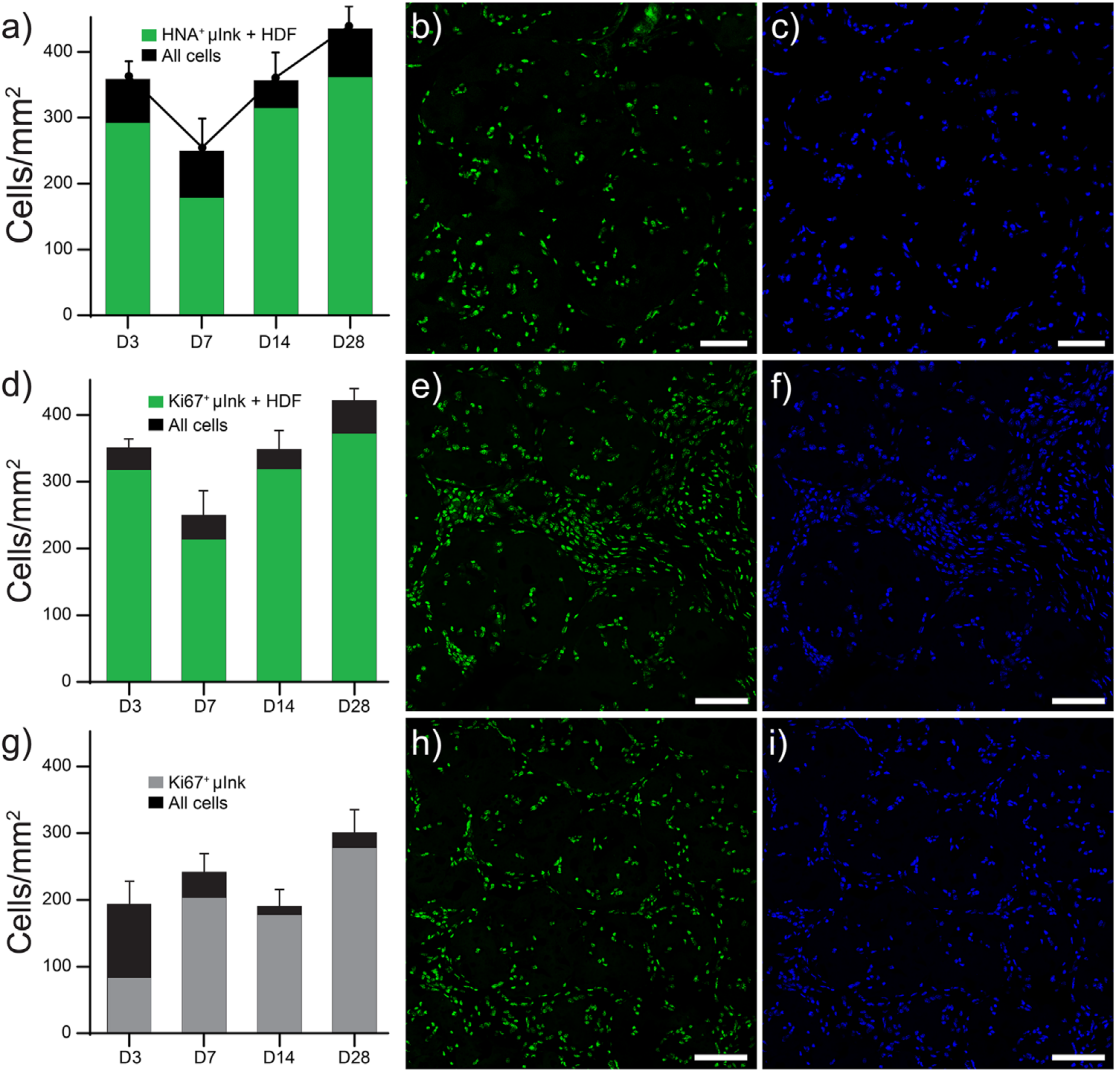
Quantification of cells in implants using a) Human Nuclear Antigen (HNA) in µInk + HDF constructs to distinguish transplanted (HNA^+^, green) human cells from infiltrating host cells (black) over time. b‐c) Representative image corresponding to a) of HNA^+^ and DAPI (blue) stained cells, respectively. d) Proliferating cells (Ki67+, green) and total cells (black) in µInk + HDF constructs. e‐f) Representative images corresponding to d) of Ki67^+^ and DAPI (blue) stained cells, respectively. g) Proliferating and total cells in acellular µInk constructs. h‐i) Representative images corresponding to g) of Ki67^+^ and DAPI (blue) stained cells, respectively. HNA staining is not applicable in the acellular µInk group and is thus not shown. Scale bars: 25 µm.

Infiltration of endogenous mouse cells into the acellular µInk implants was evident already at day three post‐implantation and the cell number (normalized per area) increased over time. In the µInk + HDF group, the number of mouse cells (calculated as total cells minus HNA‐positive human cells; **Figure** **10**a, black bar segments) increased from day 3 to day 7, followed by a decrease on day 14, before increasing again by day 28. This non‐linear pattern may reflect dynamic processes of immune response and tissue remodeling. The difference in total cell numbers between µInk + HDF and the acellular µInk group across timepoints suggests a growing population of transplanted cells in the former. NB, only the µInk + HDF group is shown in panels a–c of Figure 10, as HNA staining is used to distinguish human cells from host mouse cells. Since human‐specific staining cannot be applied to the acellular µInk group, only one group is shown for this analysis. The distribution of nuclei in the implants was, however, different between groups at early timepoints. While infiltrated mouse cells seemed to follow contours and pores of the PGM‐BCN, transplanted HDFs in the µInk + HDF group were to a larger extent present within the bulk of the PGM‐BCN. The nuclei of infiltrating cells were ≈20% smaller on average (data not shown), indicating species differences and differences in cell types. We used human nuclear antigen (HNA) to determine the proportion of transplanted (human) cells versus infiltrating (mouse) cells. Whereas the µInk group without transplanted HDFs was negative for HNA, the majority of nuclei detected in the µInk + HDF group were HNA positive (i.e., of human origin). In the µInk + HDF group, the number of mouse cells was roughly one fourth of the number of infiltrated cells in the µInk group. The number of infiltrating mouse cells in the µInk + HDF constructs increased over time except for a significant decrease on day 7. We hypothesize that the cellular infiltration during the initial days after transplantation is attributable to white blood cells enacting injury response. The subsequent increase in cell infiltration may be attributable to events related to remodeling and tissue integration, related to stromal cell division and neovascularization.^[^ ^64^, ^65^ ^]^ The difference in total number of cells between µInk + HDF and the cell‐free µInk constructs across timepoints suggests a viable and growing population of transplanted cells. The decrease of infiltrated cells in the µInk constructs on day 14 (Figure 10c), as compared to the decrease seen on day 7 in the µInk + HDF group suggest that the transplanted HDFs contributed to faster tissue regeneration.

We then proceeded to investigate the proliferative state of transplanted cells using Ki67 immunostaining. The increase in numbers of human cells over time indicates active proliferation, which is consistent with the observation of the µInk + HDF constructs in vitro, which was further supported by positive Ki67 staining for most of the cells in the implants. This shows that transplanted HDFs were proliferative (not in G0) and implies sustained viability of cells within the constructs. Infiltrating cells were also mostly proliferative, although we did not determine the contribution of proliferation over migration to the increasing mouse cell numbers. Although some degree of homogeneity among transplanted cells is a reasonable assumption in this timeframe, the infiltrating cells will be of several types and cell types will vary with time. The athymic background of the model sets limits on the generalizability of the biological cascade, but further markers could be investigated to ascertain cellular phenotypes and clarify the situation.

### 2.10 Expression of Extracellular Matrix Proteins in Transplanted Constructs

**Figure 11:**
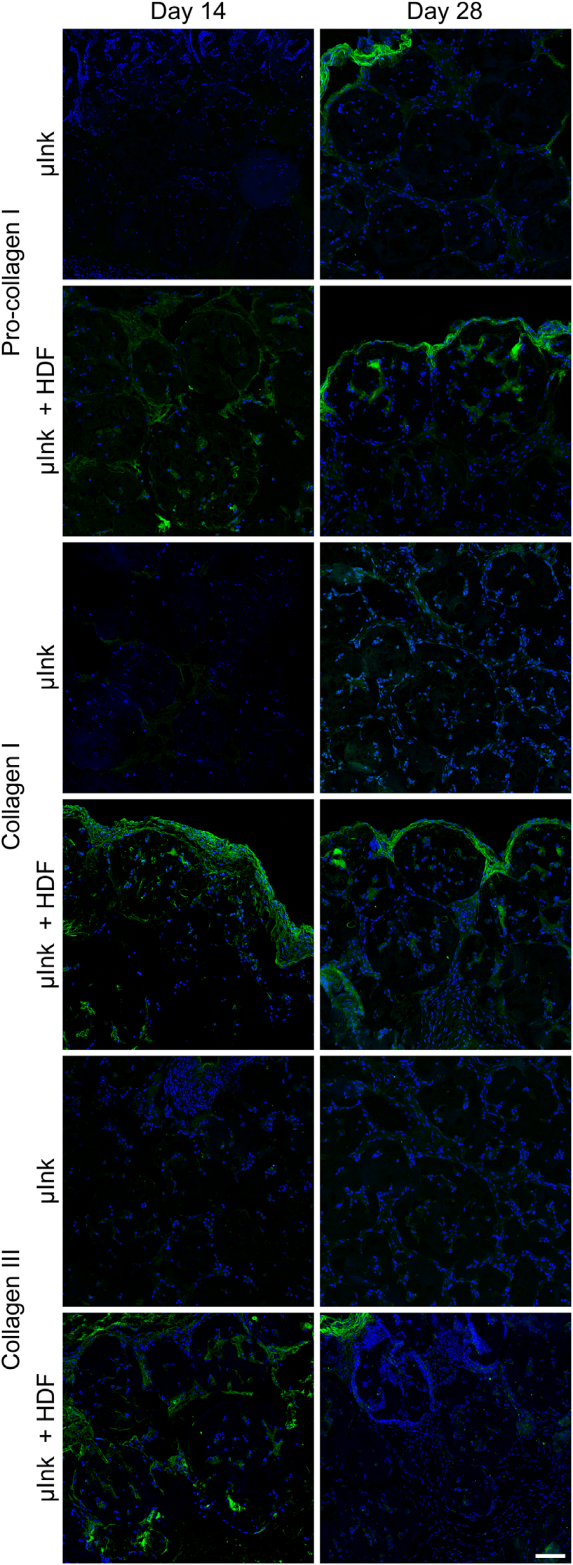
Immunofluorescence images of representative tissue sections from μInk and μInk + HDF constructs at day 14 and day 28, stained for pro‐collagen I, collagen I, and collagen III (green). DAPI (blue) was used as a nuclear counterstain. Negative control images were omitted from the figure. Scale bar: 50 µm.

Staining for pro‐collagen I, collagen I, and collagen III provide critical insights into the process of collagen synthesis, maturation, and remodeling. Expression of pro‐collagen I serves as a marker for early collagen synthesis.^[^ ^66^ ^]^ Collagen III is synthesized during the early stages of wound healing and can thus aid in the assessment of the tissue remodeling processes to identify areas undergoing active tissue repair.^[^ ^67^ ^]^ Collagen III is then replaced by collagen I, being the predominant type of collagen in normal uninjured dermis. Staining for collagen I is thus important to assess the later stages of wound healing^[^ ^68^ ^]^ and allows for visualization of the deposition and organization of mature collagen fibers. Pro‐collagen I was expressed on day 14 in the µInk + HDF group but not in the µInk group (**Figure** **11**), illustrating that the transplanted cells maintained their ECM‐producing phenotype as observed during in vitro culture. It was expressed in both groups by day 28, showing that collagen production had a head start in the group with HDFs. Collagen III was not expressed in the µInk group throughout the experiment but was expressed abundantly in the µInk + HDF group on day 14, with a decreased expression by day 28. This is in line with physiological collagen III synthesis during wound healing. Collagen I was present at both timepoints in the µInk + HDF group, pointing to ongoing replacement of collagen III by collagen I, mimicking the remodeling/maturation and acquisition of normal dermal tissue architecture. Collagen expression in the µInk+HDF group was also prominent both inside and surrounding the PGM, further illustrating a mature dermal phenotype of the transplanted cells.

**Figure 12:**
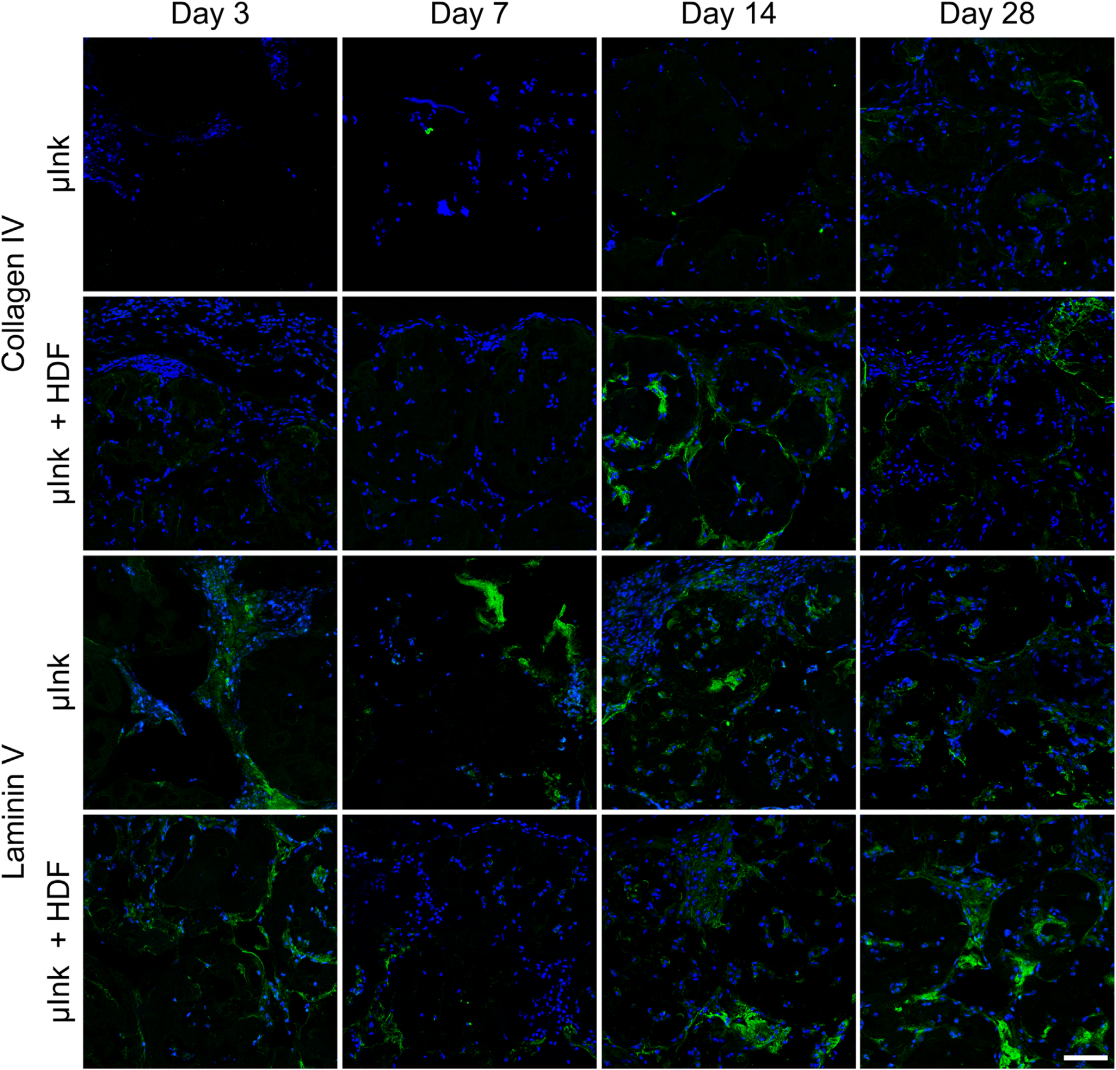
Immunofluorescence images of representative tissue sections stained for collagen IV and laminin V (green). DAPI (blue) was used as a counterstain. Negative control images were omitted from the figure. Scale bar = 50 µm.

Collagen IV and Laminin V are key components of the network‐forming ECM and are prominent constituents of the basal membrane.^[^ ^69^ ^]^ The presence of these proteins is relevant in assessing the establishment of a supportive microenvironment. Sparse expression of collagen IV can be seen for the µInk group (**Figure** **12**). However, for the µInk + HDF group, expression of collagen IV was seen by day 14, which then decreased by day 28. Laminin V was expressed throughout all timepoints in both groups. Co‐presence of collagen IV and laminin V indicate a microenvironment conducive to tissue repair and regeneration, playing a key role in the reconstitution of a basement membrane. Thus, both the routine (Figure 9) and immunohistology (Figures 11 and 12) staining show that there is substantially more ECM being produced in the µInk + HDF group compared to in the cell‐free µInk group, after transplantation at all time points.

Vascular endothelial marker CD31 is a cell surface protein, which is predominantly expressed by endothelial cells, thus serving as a marker for vascular structures.^[^ ^70^ ^]^ α‐SMA is a cytoskeletal protein, which is associated with smooth muscle cells. Staining for α‐SMA may be used to identify and assess presence of vascular smooth muscle cells in newly formed blood vessels.^[^ ^71^ ^]^

**Figure 13:**
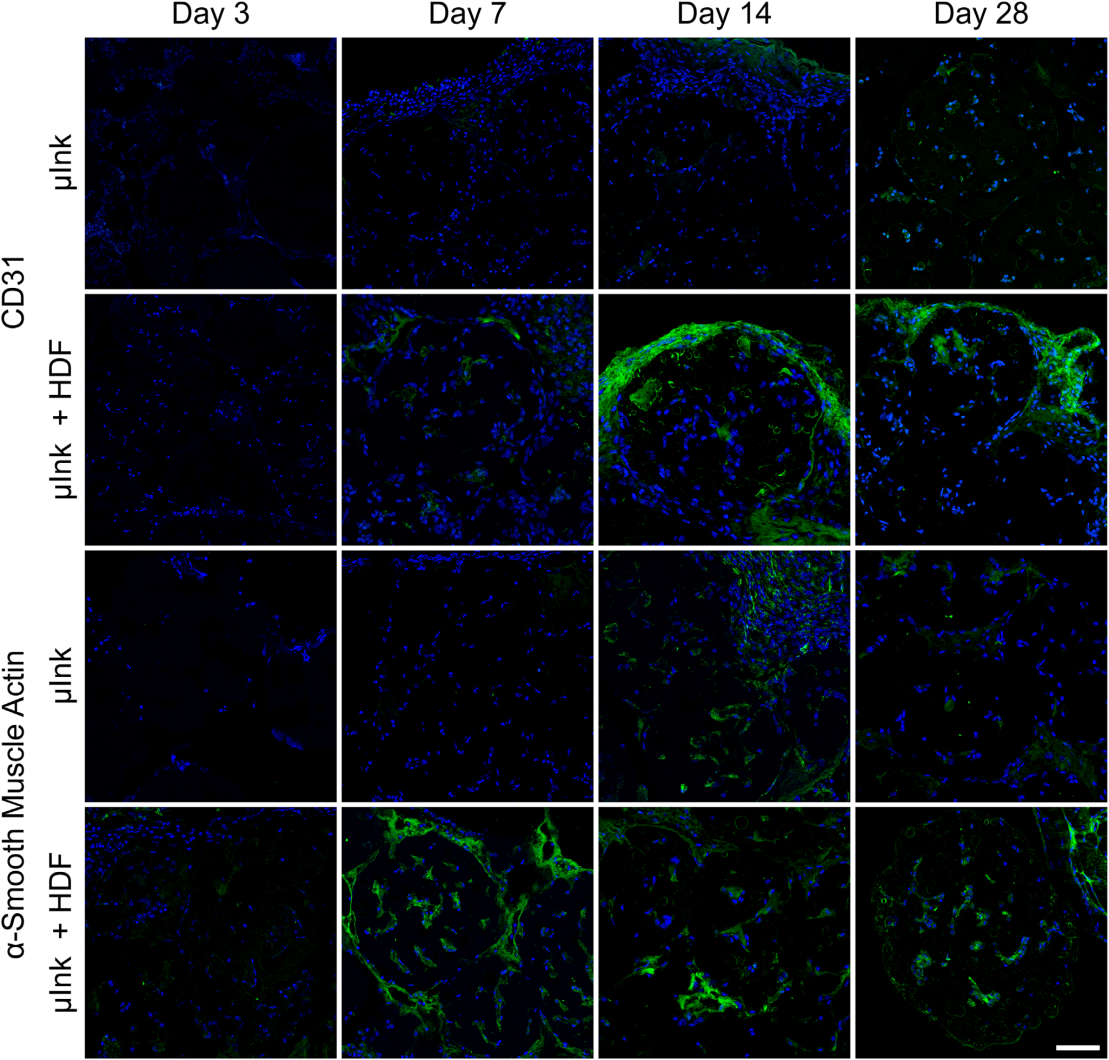
Immunofluorescence images of representative tissue sections stained separately for CD31 and α‐SMA (green) in µInk and µInk + HDF groups at 3, 7, 14, and 28 days. DAPI (blue) was used as a nuclear counterstain. Note that CD31 and α‐SMA were visualized in distinct sections and do not represent co‐localization. Scale bar = 50 µm.

The combination of CD31 and α‐SMA contributes to the assessment of vascularization within implanted µInk and µInk + HDF. Immunofluorescent staining showed an abundant expression of CD31 on day 14 and 28 for the implanted µInk + HDF, but sparse expression at any timepoint for the implanted µInk without cells. α‐SMA was expressed from day 7 and onward in the µInk + HDF group and was expressed from day 14 in the µInk group (**Figure** **13**). Positive staining for CD31 and α‐SMA in the µInk + HDF group, contrasted with the negative staining or delayed expression in the µInk group suggesting that the implanted HDF cells contributed to enhanced tissue integration and recruitment of endothelial cells.

Importantly, CD31 and α‐SMA were stained in separate sections and not as a co‐localized marker pair. Thus, spatial overlap between CD31 and α‐SMA cannot be inferred from these images. Additionally, α‐SMA is not specific to vascular smooth muscle cells and can also indicate the presence of activated myofibroblasts, which are common in tissue remodeling contexts. The observed differences in fluorescence intensity over time—especially the decrease in α‐SMA staining at later time points—do not necessarily imply cell death or vascular loss, but may instead reflect temporal changes in the role and distribution of myofibroblasts during matrix remodeling and integration. Despite variability in signal intensity, the earlier onset and stronger expression of CD31 and α‐SMA in the µInk + HDF group suggest an accelerated and enhanced vascularization process compared to the acellular µInk group. Negative controls of all immunohistochemical staining can be viewed in Figure S9 (Supporting Information).

## 3 Conclusions

In summary, we show a novel strategy for rapid and efficient expansion of harvested HDFs on PGMs and fabrication of an ultra‐high‐cell density biphasic granular bioink for dermal tissue regeneration. Harvested HDFs were seeded and expanded on PGMs in a bioreactor prior bioink formulation. The HDF laden PGMs where then combined with a HA‐based hydrogel, cross‐liked by bioorthogonal SPAAC, for generating a biphasic and shear‐thinning bioink (µInk). The µInks demonstrated excellent printability. Robust free‐standing constructs could be 3D bioprinted that showed high cell viabilities and that were stable for weeks under culture conditions. Both the PGM and the HA components in the constructs could be degraded by proteases and hyaluronidases, respectively, enabling efficient cell‐mediated remodeling after transplantation. The HDFs remained proliferative, synthesized all the relevant dermal ECM proteins, and initiated remodeling of the constructs to generate dermal tissue units. Subcutaneous dorsal implantation of 3D bioprinted µInk containing HDFs showed enhanced tissue integration and construct remodeling, compared to µInk without HDFs. The transplanted HDFs remained viable and proliferative, while expressing stromal and membranous ECM molecules and showing a regenerative dermal phenotype throughout the 28‐day experiment. Moreover, the HDFs promoted increased neovascularization of the transplanted constructs together with production of dermal‐epidermal junction constituents further suggest a shift toward regeneration as opposed to scar formation. Continued experiments will show the phenotypic dynamics of transplanted cells in a porcine wound model, which has high homology to human wound healing to explore the mechanisms for dermal tissue regeneration in more detail and to lay the foundation for clinical testing of the µInk technology. In summary, the use of µInk for biofabrication is a promising methodology for translating advanced dermal tissue engineering constructs to clinical use.

## 4 Experimental Section

### Materials

Hyaluronan (100–150 kDa) and 4‐arm PEG azide (P(N_3_)_4_), 10 kDa) were procured from Lifecore Biomedical (Minneapolis, USA) and Creative PEG works (North Carolina, USA), respectively. Porous gelatin microcarriers (PGMs), Cultispher‐S, were obtained from Percell Biolytica AB, Åstorp, Sweden. Gelatin (Type A, gel strength 300), (1R,8S,9s)‐Bicyclo [6.1.0] non‐4‐yn‐9‐yl methyl N‐succinimidyl carbonate (BCN‐NHS) and all other chemicals were purchased from Merck Life Sciences AB (Stockholm, Sweden) unless otherwise stated and were used without further purification.

### Ethical Approval

The present study followed the General Data Protection Regulation (GDPR) as well as the act 2002:297, which regulates bio banks and registers. All experiments involving human cells and tissues were performed under approval from the Swedish Ethical Review Authority (no. 2018/97/31). Experiments involving the use of animals were approved by the Ethics Committee for Animal Experiments at Sahlgrenska University Hospital (University of Gothenburg, Gothenburg, Sweden: Dnr 5.8.18‐09289/2020) and were conducted in accordance with institutional, national, and European guidelines and regulations. All animal experiments were performed at the core facility for experimental biomedicine at the University of Gothenburg.

### Preparation of BCN Functionalized PGMs (PGM‐BCN)

PGMs (1 g) were hydrated in phosphate buffered saline (PBS, 1X) for 2 h at RT. After complete aspiration of the supernatant, BCN‐NHS in DMSO (50 mL, 4 mg mL^−1^) was added and gently stirred on a platform rocker for 12 h at RT. The as‐obtained BCN modified PGMs (PGM‐BCN) were then carefully washed with PBS (≥ 10 times) and incubated in excess PBS overnight under mild stirring to ensure complete removal of unreacted BCN‐NHS. The solution was then vacuum filtered using a Steriflip centrifuge tube top filter unit (20 µm pore size, Merck) and stored at 4 °C until use. Quantification of BCN functionalization on PGM was carried out using a ninhydrin assay. Briefly, PGMs (4 mg) and freeze dried PGM‐BCN (4 mg) were hydrated with PBS (320 µL, 1X) in microcentrifuge tubes and ninhydrin (80 µL, 7.5 mg mL^−1^ in ethanol) were added. Tubes were sealed and incubated at 95 °C, until purple (≈30 min). Each solution was added to a 96‐well plate (100 µL) and the degree of functionalization (DoF) was then estimated by comparing color intensity. Fluorescence measurements were also carried out to quantify BCN groups by reacting PGM‐BCN (25 mg) with fluorescent Sulpho‐Cy5 azide dye (1 mM, 3 h) that undergoes spontaneous click reaction with the BCN groups. Once the unbound dye was washed off thoroughly with PBS, 10 mg of the Cy5 labelled PGM‐BCN was digested with collagenase (400 µL, 2.5 mg mL^−1^) in HEPES buffer supplemented with Ca^2+^ (5 mM) and further diluted one thousand times. Moles of BCN groups per mg of PGM‐BCN were then estimated by comparing the intensity of fluorescence emission of Cy5 conjugated PGM‐BCN (n = 3) to that of the standard curve of Cy5.

### Synthesis of BCN Functionalized HA (HA‐BCN)

HA‐BCN was synthesized by EDC/NHS coupling as described in more detail elsewhere.^[^ ^37^, ^47^ ^]^ Briefly, BCN‐NH_2_ (100 mg) was dissolved in a mixture of acetonitrile and milliQ water (MQ) (5:1 v/v, 6 mL), 1‐Hydroxybenzotriazole hydrate (83 mg, HOBt), and 1‐ethyl‐3‐(3‐dimethylaminopropyl) carbodiimide hydrochloride (236 mg, EDC). The solution was further mixed with HA (500 mg) dissolved in MES buffer (40 mL, 100 mM, pH 7), and stirred overnight. The solution was dialyzed against acetonitrile (10%, 24 h), MQ (7 days), and adjusted to pH 6.5. Finally, the solution was diluted with MQ (1:1) and freeze dried to obtain HA‐BCN. ^1^H‐NMR analysis was used to determine the degree of BCN substitution to HA backbone as described elsewhere.^[^ ^72^ ^]^

### Fabrication of Biphasic Granular Hydrogel (µInk)

For fabrication of the µInk, PGM‐BCN (100 mg) was mixed sequentially with HA‐BCN (25 µL, 2%), gelatin (8.4 µL, 1%), and P(N_3_)_4_ (8.4 µL, 6%). The µInk was then incubated at 37 °C for 3 h in a humid environment for complete gelation and stored at 4 °C until further use.

### Morphological Evaluation and Porosity Analysis

The surface morphology and cross‐sections of the PGM, PGM‐BCN and µInk were analyzed using both scanning electron microscopy (SEM, LEO Gemini 1550, Carl Zeiss, Oberkochen, Germany) and laser scanning confocal microscopy (LSM 780, Carl Zeiss, Oberkochen, Germany). For SEM analysis, samples were dehydrated using a graded ethanol series (0%, 10%, 20%, 40%, 60%, 80%, 90%) for 10–15 min each, followed by treatment with hexamethylsilazane (100%) for 30 min. Samples were vacuum dried, sputter coated with platinum for 15 s and imaged at an accelerating voltage of 5 kV. For confocal imaging, biphasic µInk with Cy3 labelled HA‐BCN and Cy5 labelled PGM‐BCN was constructed. Images were acquired using either 10× or 20× objectives. Sample porosity of the sample was analyzed using Fiji v1.54i (ImageJ, NIH, Maryland, USA) from confocal Z‐stacks (≈140 µm) taking n = 10 slices as per the protocol reported by Caprio et al.^[^ ^28^ ^]^ Porosity (%) was defined as area of pores in a slice expressed as a percentage of total slice area.

### Oscillatory Rheology

The mechanical properties of the hydrogels were studied using oscillatory rheology (Discover HR‐2, TA instruments, New Castle, Delaware, USA) at 37 °C. The µInks were prepared by mixing PBS swollen PGM‐BCN (100 mg), HA‐BCN (25 µL, 2%), gelatin (8.4 µL, 1%), and P(N_3_)_4_ (8.4 µL, 6%) and were then subjected to time sweeps at 1% strain and 1 Hz frequency on a 20 mm parallel plate geometry for 2 h at 37 °C prior to more in depth mechanical property assessment.^[^ ^73^ ^]^ A gap size of 800–900 µm and an axial force between 0.01–0.02N were used for all tests. Amplitude sweeps were performed between 0.1% to 100% strain at 1 Hz frequency. Frequency sweeps were conducted between 0.1–0 Hz at 1% strain. High strain and low strain cyclic sweeps were performed at 500% and 0.5% strain respectively at 1 Hz frequency.

### Enzymatic Degradation of µInk

Small sample discs of µInk were prepared by mixing hydrated PGM‐BCN (30 mg), HA‐BCN (7.5 µL, 2%), gelatin (2.5 µL, 1%) and P(N_3_)_4_ (2.5 µL, 6%) sequentially and incubating at 37 °C for 3 h in a humid environment. The degradation behavior in collagenase was studied using sample discs prepared using Cy3 conjugated PGM‐BCN, while Cy5 conjugated HA‐BCN was used to prepare sample discs to study degradation in hyaluronidase. Discs were transferred to 4 mL cuvettes where 4‐(2‐hydroxyethyl)‐1‐piperazineethanesulfonic acid (2 mL 30 mM, HEPES buffer, pH 7) was supplemented with Ca^2+^ (5 mM). Fluorescence emission intensities at 562 nm (λ_ex_ 540 nm, Cy3) and at 663 nm (λ_ex_ 640 nm, Cy5) were recorded after 2 h incubation at 37 °C, corresponding to time zero. The buffer was then replaced with collagenase (2 mL, 0.05 mg mL^−1^, Type 1) for discs prepared with Cy3‐PGM‐BCN and hyaluronidase from bovine testes (2 mL, 0.05 mg mL^−1^, Type 1‐S, 400–1000 units mg^−1^ solid) for discs prepared with Cy5‐HA‐BCN. After incubation at 37 °C, fluorescence emission intensity was recorded at definite time intervals up to 24 h after which collagenase (20 µL, 10 mg mL^−1^) and hyaluronidase (20 µL, 10 mg mL^−1^) were added to the respective cuvettes to completely degrade samples. The fluorescence emission intensity was noted to determine the percentage of the degraded hydrogel.

### 3D Bioprinting

3D extrusion bioprinting was performed using a Cellink BioX (Cellink AB, Gothenburg, Sweden). Briefly, PGM‐BCN (300 mg), HA‐BCN (75 µL, 2%), gelatin (25 µL, 1%), and P(N_3_)_4_ (25 µL, 6%) were mixed well. The µInk was then loaded into a printing cone (20 G) and incubated at 37 °C in a humid environment for 60–75 min. The cone was subjected to quick centrifugation using a mini centrifuge to remove any air bubbles and then attached to a syringe inside the bioprinter to enable printing of different self‐standing 3D structures – cylinder (5 mm × 10 mm), square prism (5 mm × 5 mm × 5 mm) and 5‐pointed star (5 mm × 5 mm) were printed with a tip travel speed of 3 mm s^−1^ and a nozzle pressure of 25–32 kPa. Fusion 360 (version 2.0.18950×86_64, Autodesk, USA) was used to create 3D models, while slicing and exporting STL files were performed using Ultimaker Cura (version 5.7.1, Ultimaker B. V, Netherlands).

### Isolation of Fibroblasts

Primary human dermal fibroblasts (HDFs) were obtained from healthy patients undergoing routine abdominoplasty at Linköping University Hospital, Linköping, Sweden. Briefly, cells were isolated by mechanical dissection and enzymatic digestion of the dermis under sterile conditions. Skin samples were repeatedly washed in sterile PBS and subcutaneous fat was mechanically removed. The remaining dermis was dissected into millimeter‐sized pieces and placed in Dulbecco's modified Eagle's medium (DMEM; Gibco Thermo Fisher Scientific, Paisley, UK) with collagenase (Gibco Thermo Fisher Scientific, 165 U mL^−1^) and dispase (Gibco Thermo Fisher Scientific, 2.5 mg mL^−1^) and incubated at 37 °C, 5% CO_2_, and 95% humidity for 24 h. After enzymatic digestion, the suspension was centrifuged at 200 *g* for 5 min and the resulting cell pellet was resuspended in HDF medium (HDFM; DMEM containing 10% fetal calf serum, 50 U mL^−1^ penicillin, and 50 µg mL^−1^ streptomycin). Cells were seeded into 75 cm^2^ culture flasks (Falcon, Corning Inc; Corning, NY) in HDFM and kept in an incubator at 37 °C, 5% CO_2_, and 95% humidity. Medium was changed three times per week and cells were passaged 1:3 when confluent.

### Cell Seeding on PGM‐BCN

PGM‐BCN submerged in PBS (20 mL) was autoclaved and washed with PBS once and HDFM twice. PGM‐BCN and HDFM (50 mL) were transferred to a spinner flask (MCS‐104S Biological Stirrer, Bio‐Techne, Minneapolis, US). HDFs were detached from the culture flasks using versene and trypsin (2.5%) (1:1, Gibco Thermo Fisher) and 3 million HDFs were added to the spinner flask containing PGM‐BCN. Flasks were intermittently stirred at 35 rpm for 5 min every hour for the first 24 h. The next day, HDFM (50 mL) was added to the spinner flask, and the stirring was set to continuous. Half of the medium was changed every other day.

### Proliferation Assay

Every 24 h, cell laden PGM‐BCN (10 mL) was transferred to a falcon tube. Cell medium was removed, leaving 1 mL containing PGM‐BCN, which was carefully washed with PBS 3 × 5 min. PBS was then removed and the PGM‐BCN‐HDF were transferred to a microscopy slide and mounted using ProLong Glass with NucBlue (Thermo Fisher Scientific). Cells per PGM‐BCN were counted in tiled images using image processing software Fiji v1.54i (ImageJ, NIH).

### Fixation and Visualization of Cells on PGM‐BCN

Cell‐laden PGM‐BCN were transferred from spinner flask to confocal dishes (35 mm glass‐bottom dishes) and washed with PBS twice. Cells were fixed by adding formaldehyde (4%, 2 mL, Thermo Fisher Scientific) and incubated for 20 min. PGM‐BCN were washed with PBS and used for subsequent staining.

### Immunocytochemical Analysis

Fixed cell‐laden PGM‐BCN were washed with PBS containing Tween (0.1% PBT; Thermo Fisher Scientific) and incubated for 30 min in bovine serum albumin (2.5%, BSA; Sigma Aldrich). Samples were then washed and incubated with primary antibodies (Table S1, Supporting Information) diluted in PBS at 4 °C overnight. PGM‐BCN were washed in PBT (0.1%) 3 × 5 min and incubated with secondary antibodies and 4′,6‐diamidino‐2‐phenylindole (DAPI, 10 µg mL^−1^) for 1 h at ambient temperature. Samples were then washed with PBS 3 × 10 min, mounted with ProLong Glass Antifade Mountant (Invitrogen) and imaged using a confocal microscope (LSM700, Zeiss) and a fluorescence microscope (DMi8, Leica).

### Semi‐Quantitative Real Time PCR

HDF‐laden PGM‐BCN were extracted from the spinner flask and dissolved using versene/trypsin (1:1) as described above. Cells were centrifuged at 200 *g* for 5 min and the supernatant removed. RNA from the cell pellet was isolated using a Total RNA isolation kit (Applied Biosystems, Thermo Fisher Scientific) according to manufacturer's instructions. The yield (ng µL^−1^) of the eluted RNA sample was measured spectrophotometrically using a NanoDrop (ND‐1000, Thermo Fisher). Complementary DNA was synthesized using High‐Capacity RNA‐to‐cDNA kit (Applied Biosystems) in a thermal cycler (PCR Thermal Cycler 2720, Applied Biosystems), with a target of 1000 ng of template RNA per sample. The cDNA was stored at ‐20 °C until further use. The thermowell PCR 96‐well fast plate (Corning Inc.) was prepared with qPCR master mix (TaqMan Fast Advanced Master Mix, Applied Biosystems), TaqMan gene assays (Table S2, Supporting Information), nuclease‐free water and cDNA to a total volume of 10 µL. The plate was centrifuged at 365 *g* for 1 min and placed in a real‐time thermal cycler (QuantStudio 7 Flex, Applied Biosystems). Data was analyzed using the 2^−ddCq^ method^[^ ^74^ ^]^ to obtain gene expression fold changes.

### Bioprinting with Cells

HDF cultured on PGM‐BCN for three days were used to prepare µInk as described in section 2.11. Briefly, HDF‐laden PGM‐BCN (1800 mg), HA‐BCN (450 µL, 2%), gelatin (150 µL, 1%), and P(N_3_)_4_ (150 µL, 6%) were mixed, loaded into a cartridge fit with a 20 G cone and incubated at 37 °C in a humid environment for 1 h 15 min. After a quick centrifugation of the cartridge, 5×2 mm solid cylinders were bioprinted using a nozzle pressure of 25–30 kPa. Printed constructs were incubated at 37 °C for 1 h 15 min for complete crosslinking followed by incubation in HDFM at 37 °C for 48 h before implantation, and up to 72 h for assessment of viability min vitro.

### Cryopreservation of µInk + HDF Constructs

Cell‐laden bioprinted constructs were placed in formaldehyde (4%) for 20 min and transferred to 30% sucrose until saturated. Constructs were then embedded in OCT Compound (Optimal Cutting Temperature Compound, Thermo Fisher Scientific) in cryomolds (Peel‐A‐Way, Thermo Fisher Scientific) and snap‐frozen in 2‐methylbutane. Frozen samples were stored at –80 °C until sectioning, which was performed using a CM1950 Cryostat (Leica). Sections were mounted on microscopy slides for subsequent staining.

### Phalloidin Staining

Frozen sections were thawed at room temperature for 10 min, washed with PBS and incubated in Triton‐X (0.1%, Thermo Fisher Scientific) for 5 min at RT. Sections were then washed with PBS twice and blocked with BSA (2.5%) for 20 min. BSA was removed and sections were incubated in Phalloidin (Alexa Fluor 594 Phalloidin, A12381, Thermo Fisher Scientific) and DAPI (10 µg mL^−1^) for 20 min at RT. Sections were subsequently washed in PBS, allowed to air dry and mounted with ProLong Glass (Thermo Fisher Scientific). Samples were visualized using a fluorescence microscope (DMi8, Leica).

### Viability Assessment

Zombie Red (BioLegend, San Diego, US) was diluted 1:1000 in PBS, added to the cell‐laden PGM‐BCN and incubated in the dark for 20 min. Constructs were washed with HDFM, fixed using formaldehyde (4%) and incubated for 20 min. After fixation, constructs were washed in PBS and incubated in DAPI (10 µg mL^−1^) for 30 min. Constructs were imaged using a confocal microscope (LSM700, Zeiss) and a fluorescence microscope (DMi8, Leica).

### In Vivo Implantation

Female Balb/c mice (aged 9 weeks; Scanbur, Karlslunde, Denmark) were anesthetized by isoflurane (4% induction, 2% maintenance; air flow: 2 L min^−1^) in a gas chamber. A dorsal incision was made in the skin using a scalpel and a 3D bioprinted construct (cell‐free or cell‐laden; n = 24 per group) was implanted subcutaneously (Figure S10, Supporting Information). The incision was closed using polyglactin sutures (5‐0 Vicryl Rapide; Ethicon Inc., Raritan, NJ, USA) and dressed using sterile wound tape. No antibiotics were used in the study. On days 3, 7, 14, and 28, animals (n = 6 per group) were euthanized by cervical dislocation and the constructs were exposed by surgical dissection and optically assessed after which they were explanted. All explanted constructs were immediately fixed in buffered formaldehyde solution (6%, Histofix; HistoLab, Gothenburg, Sweden) for 24 h at RT.

### Tissue Processing and Paraffin Sectioning

At each timepoint, samples from the in vivo implantation were excised, placed in buffered formaldehyde solution (6%) overnight and submerged in PBS for 4 h. Samples were further processed using a TP1020 tissue processor (Leica Biosystems), where they underwent a series of submersions; ethanol (70%, 30 min), ethanol (90%, 1 h), ethanol (95%, 2×1 h), ethanol (99.5%, 2×30, min + 1 h), xylene (2×30 min + 1 h), and paraffin (1 h + 2 h)). Biopsies were embedded in paraffin blocks, stored at –20 °C, sectioned (8 µm thickness) using an RM2255 microtome (Leica Biosystems) and stored at RT for subsequent staining.

### Haematoxylin and Eosin Staining

Fixed sections were dried for 30 min at 60 °C and submerged in Histolab‐Clear (HistoLab Products AB, Askim, Sweden) (3 × 2 min), ethanol (99.5%, 2 min), ethanol (96%, 2 min), distilled water (1 min), Mayer's haematoxylin (HistoLab Products AB) (3 min), running tap water (4 min), eosin Y (HistoLab Products AB) (0.2%, 30 s), tap water (30 s), ethanol (96%, 30 s), ethanol (99.5%, 2 × 1 min) and Histolab‐Clear (2 × 2 min) in order. Samples were mounted on slides using Pertex mounting medium (HistoLab Products AB) and imaged using an Olympus BX51 microscope (Olympus Scientific Solutions).

### Trichrome Staining

Sections were de‐paraffinized by submersion in Histolab‐Clear (HistoLab Products AB) (2 × 10 min), ethanol (99.5%, 10 min), ethanol (96%, 5 min), ethanol (70%, 5 min), and deionized water (10 min). Slides were submerged in Bouin's solution (Sigma‐Aldrich) (15 min) preheated to 56 °C. Slides were cooled in 20 °C tap water and washed in running water (10 min), submerged in Haematoxylin (HistoLab Products AB) (5 min), washed in running water (5 min), and rinsed in deionized water. Slides were then submerged in Biebrich Scarlet‐Acid Fuchsin Solution (Sigma‐Aldrich) (5 min), rinsed in deionized water, submerged in phosphomolybdic/phosphotungstic acid solution (5%, 5 min), aniline blue solution (5 min), and acetic acid (1%, 2 min). Following rinsing in deionized water (10 s), ethanol (70%, 10 s), ethanol (96%, 10 s), ethanol (99.5%, 10 s), and Histolab‐Clear (10 s), slides were air‐dried, mounted using Pertex (HistoLab Products AB) and imaged using a BX51 microscope (Olympus).

### Immunohistochemical Analysis

Sections were de‐paraffinized as described in section 2.23. Hydrophobic wells were drawn around sections using a PAP‐pen (Liquid Blocker Pap Pen, HistoLab Products AB). Sections were incubated with BSA (2.5%) for 20 min to block non‐specific binding. BSA was then removed, and primary antibodies (Table S1, Supporting Information) were added to each section and incubated at 4 °C overnight in a dark moist chamber. Slides were washed in PBS (2 × 5 min) and incubated with secondary antibodies and DAPI at room temperature for 1 h. Following a wash in PBT (PBS + 0.1% Triton‐X100), slides were air‐dried and mounted using ProLong Glass Antifade Mountant (Thermo Fisher Scientific). Sections were imaged using a confocal fluorescence microscope (Stellaris 5, Leica).

### Statistical Analysis

Data was expressed as mean ± standard deviation (SD) except for gene expression fold‐change which was expressed as geometric mean ± geometric SD. Testing for statistically significant differences was performed using a Mann–Whitney U test, or one‐way ANOVA coupled with the Dunnett´s post‐hoc test, Holm‐Šídák test or Tukey´s HSD post‐hoc test, as indicated in respective figure legends. Sample sizes are also indicated in legends. No values were excluded from analyses. All calculations were performed using Prism 10.0.2 (Graphpad, La Jolla, US). A value of p < 0.05 was considered significant.

## Conflict of Interest

The authors declare no conflict of interest.

## Supporting information



Supporting Information

Supplemental Video 1

Supplemental Video 2

Supplemental Video 3

Supplemental Video 4

## Data Availability

Data that support the findings of this study are available from the corresponding authors upon reasonable request.
