# Supplementary material for: Biphasic Granular Bioinks for Biofabrication of High Cell Density Constructs for Dermal Regeneration
Source: Adv Healthc Mater. 2025 Jun 12;14(21):2501430. doi: 10.1002/adhm.202501430 (PMC12365623; doi:10.1002/adhm.202501430)
Supplement: Supplementary file 1 — Supporting Information [file ADHM-14-0-s002.docx]

**Supplementary Information**

**Biphasic Granular Bioinks for Biofabrication of High Cell Density Constructs for Dermal Regeneration**

Rozalin Shamasha^a,b,†^, Sneha Kollenchery Ramanathan^c,†^, Kristin Oskarsdotter^d^, Fatemeh Rasti Boroojeni^c^, Aleksandra Zielińska^a^, Sajjad Naeimipour^c^, Philip Lifwergren^c^, Nina Reustle^c^, Lauren Roberts^a^, Annika Starkenberg^a^, Gunnar Kratz^a,e^, Peter Apelgren^d^, Karin Säljö^d^, Jonathan Rakar^a,b^, Lars Kölby^d^, Daniel Aili^c,*^, Johan Junker^a,b,*^

^a^ Experimental Plastic Surgery, Department of Biomedical and Clinical Sciences, Linköping University Hospital, 581 83 Linköping, Sweden.

^b^ Center for Disaster Medicine and Traumatology, Department of Biomedical and Clinical Sciences, Linköping University Hospital, 581 83 Linköping, Sweden.

^c^ Laboratory of Molecular Materials, Division of Biophysics and Bioengineering, Department of Physics, Chemistry and Biology, Linköping University, 581 83 Linköping, Sweden.

^d^ Department of Plastic Surgery, Sahlgrenska Academy, University of Gothenburg, 405 30 Gothenburg, Sweden.

^e^ Region Östergötland, Anaesthetics, Operations and Specialty Surgery Center, Department of Hand and Plastic Surgery, Linköping University Hospital, 581 83 Linköping, Sweden**.**

^†^ Equal contribution

* Corresponding authors: [johan.junker@liu.se](mailto:johan.junker@liu.se) and [daniel.aili@liu.se](mailto:daniel.aili@liu.se)


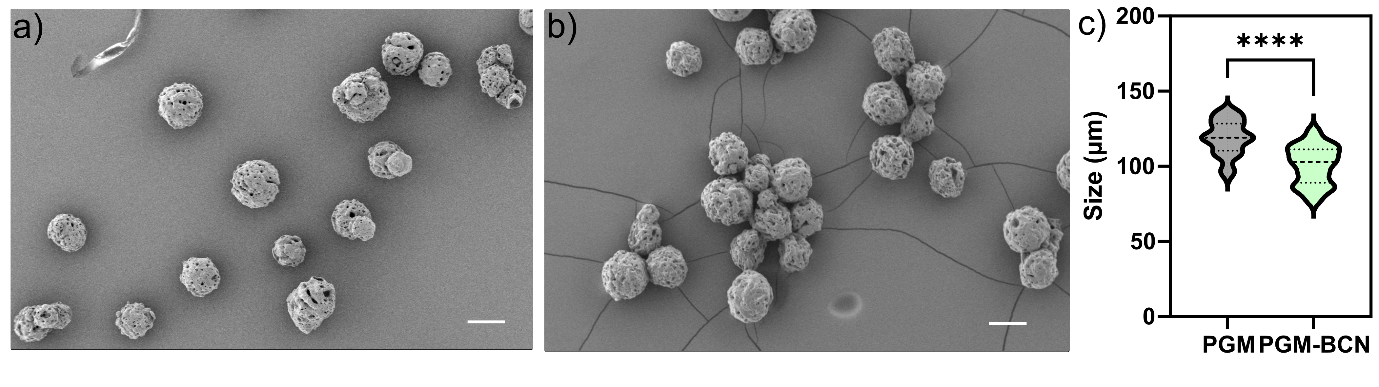


**Figure S1.** SEM images of (a) PGM and (b) PGM-BCN and c) particle size distribution of PGM and PGM-BCN. Scale bars: 100 µm


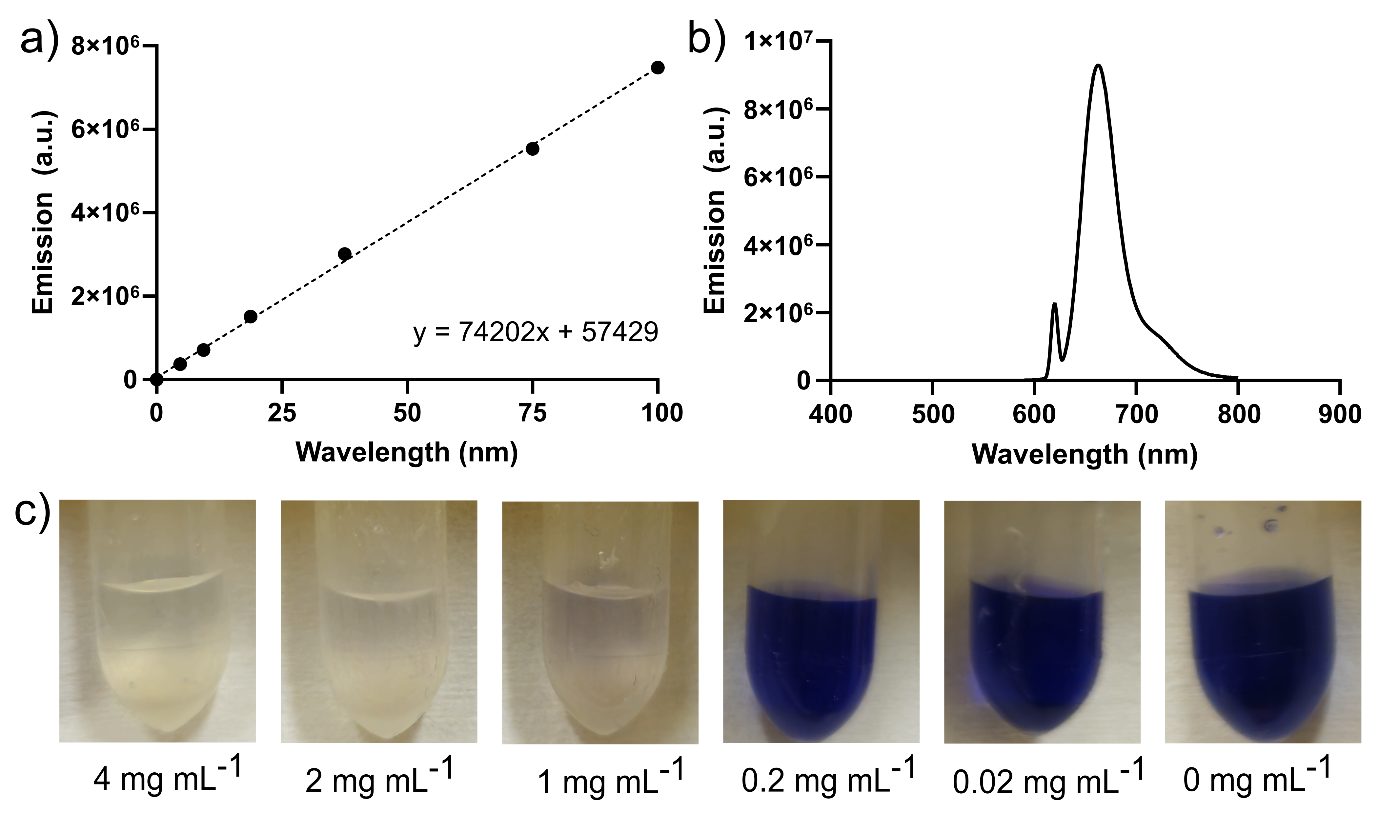


**Figure S2.** a) Photographs of Ruhemanns purple color formation by PGM-BCNs prepared with different concentration of BCN-NHS (4, 2, 1, 0.2, and 0.02 mg mL^-1^) after ninhydrin assay. The higher the BCN functionalization, the lower the intensity of purple. b) Standard curve of Cy-5. c)Emission spectra of Cy-5 labelled PGM-BCN


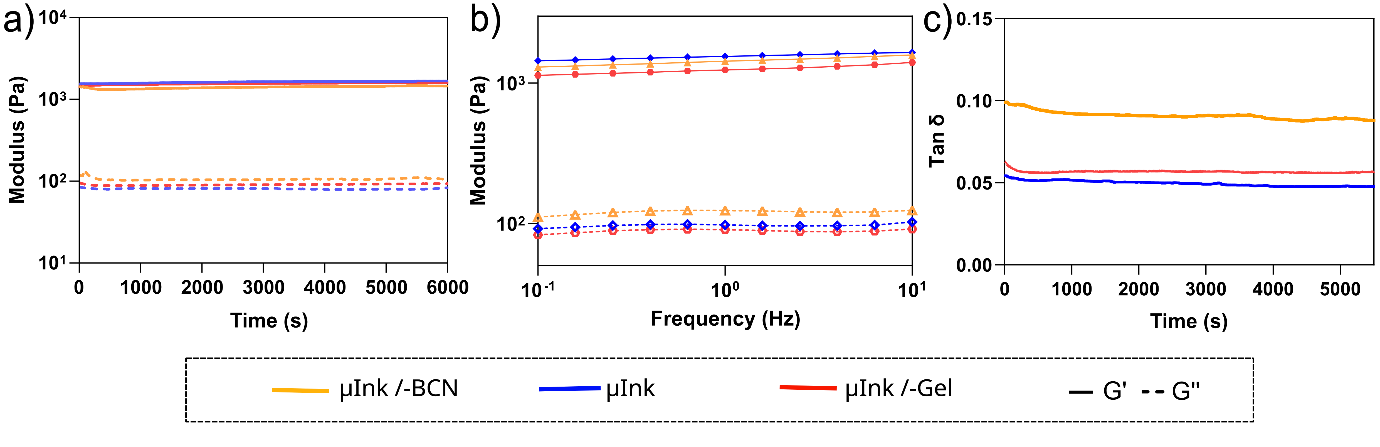


**Figure S3.** a) Time sweeps at 1% oscillation strain and 1 Hz oscillation frequency. b) Frequency sweep at 1% oscillation strain and c) Variation of tan δ over time.


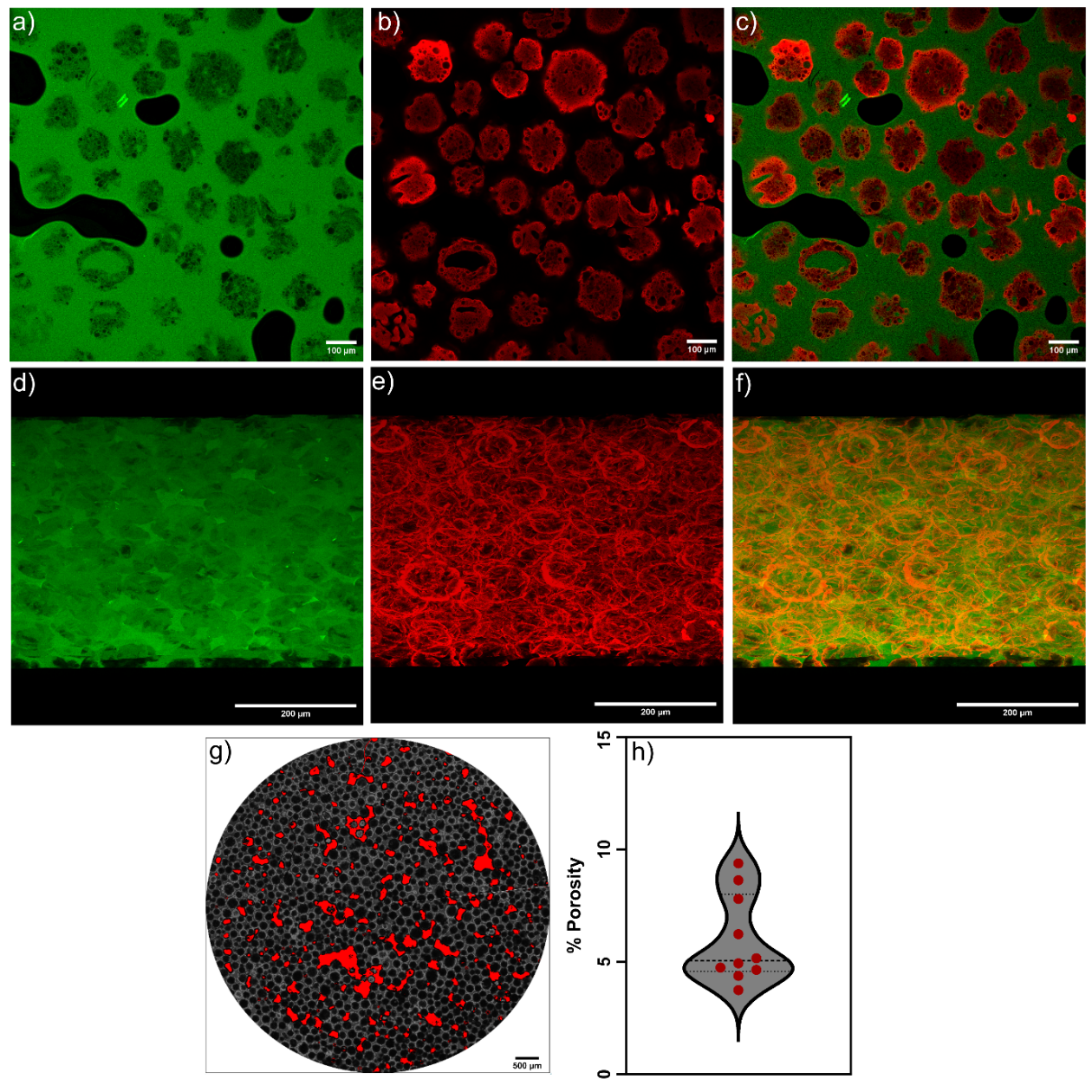


**Figure S4.** Confocal fluorescence images of the µInk with a) Cy3 labelled HA-BCN, b) Cy5 labelled PGM-BCN, c) merged image. 3D projections of d) Cy3 labelled, e) Cy5 labelled PGM-BCN, and f) merged. g) Distribution of void spaces within the stitched tile scan Figure 2g. h) Quantification of %porosity of the slices of the µInk.


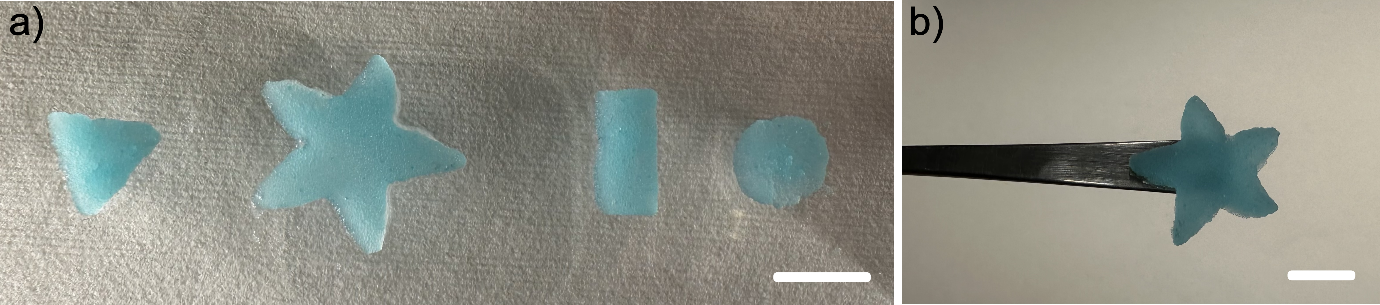


**Figure S5**. a, b) Molded µInk-based structures after 3h incubation at 37 °C. Scale bars: 5 mm


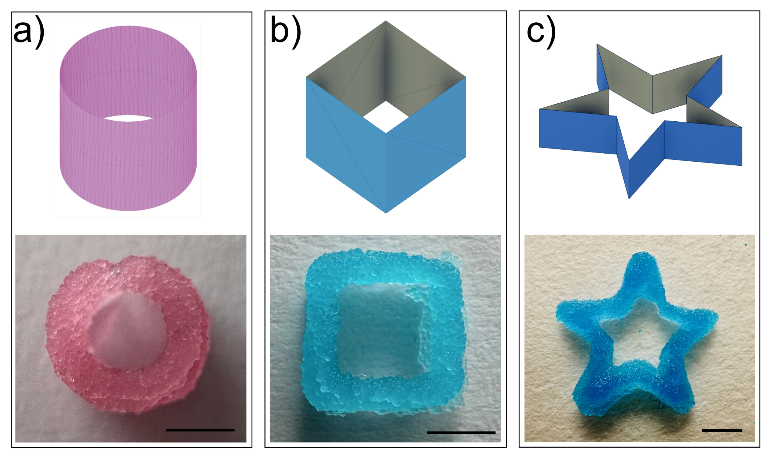


**Figure S6.** Top view of the 3D printed structures using µInk. a) Ten layered hollow cylinder, b) five layered hollow square and c) one layered five-pointed star. Scale bars: 2.5 mm

**
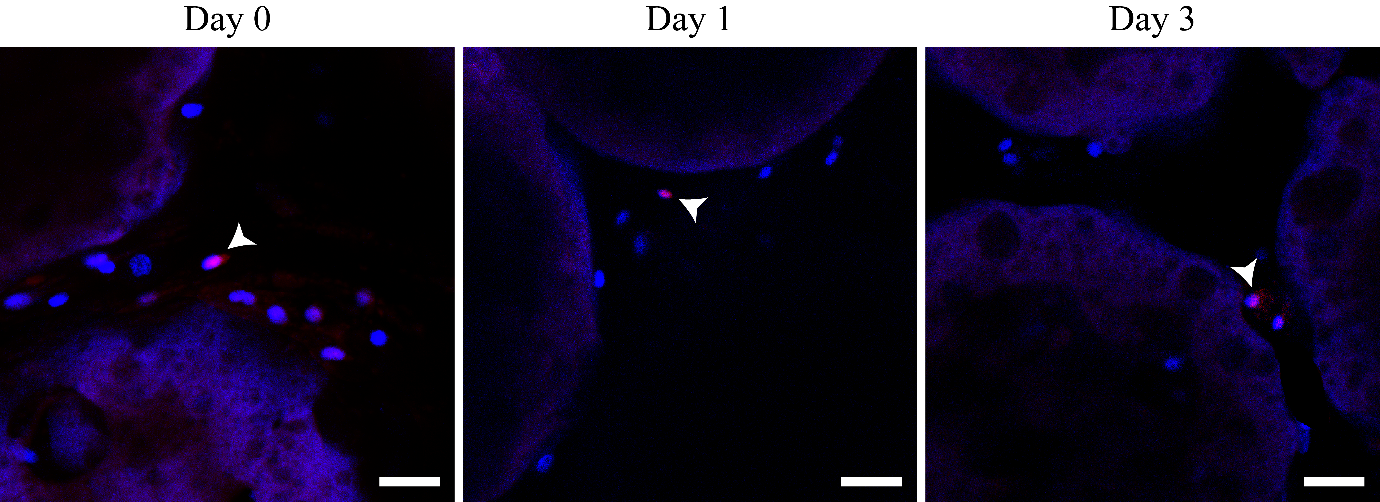
**

**Figure S7.** Representative images showing Zombie Red™ (red) and DAPI (blue) staining. Dead cells are labeled by Zombie Red™ and indicated with arrowheads. Scale bar: 25 µm.

**
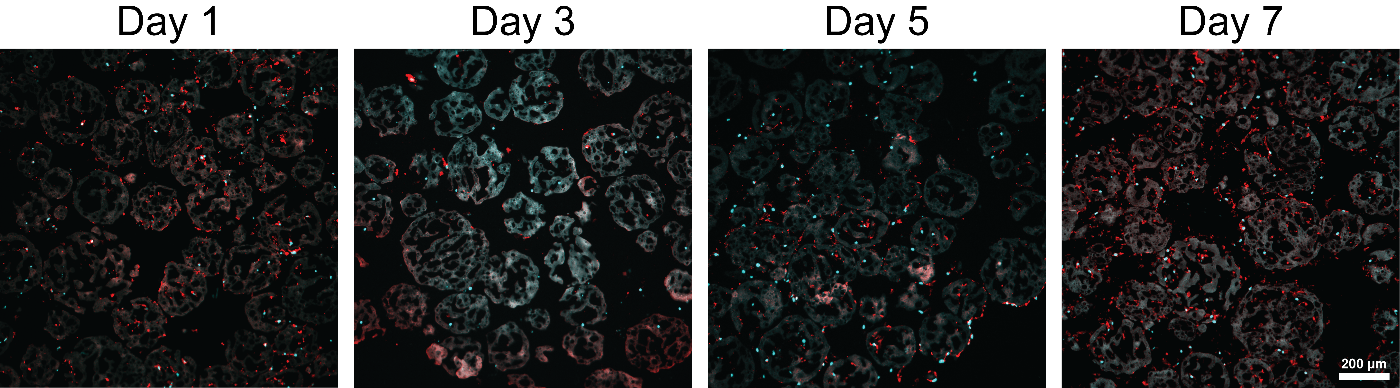
**

**Figure S8.** Representative confocal images of printed constructs stained with phalloidin (red) and DAPI (cyan). Images show optical sections (6 µm) of the construct, thus only a subset of cells are visible.


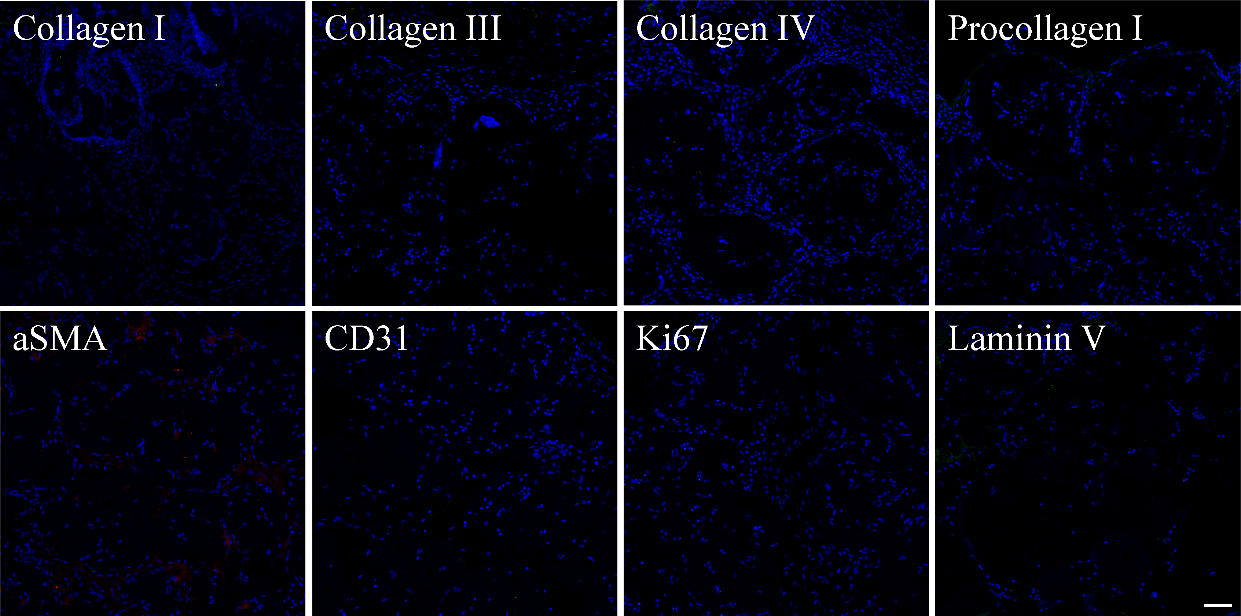


**Figure S9.** Representative images of immunohistochemical negative control samples.


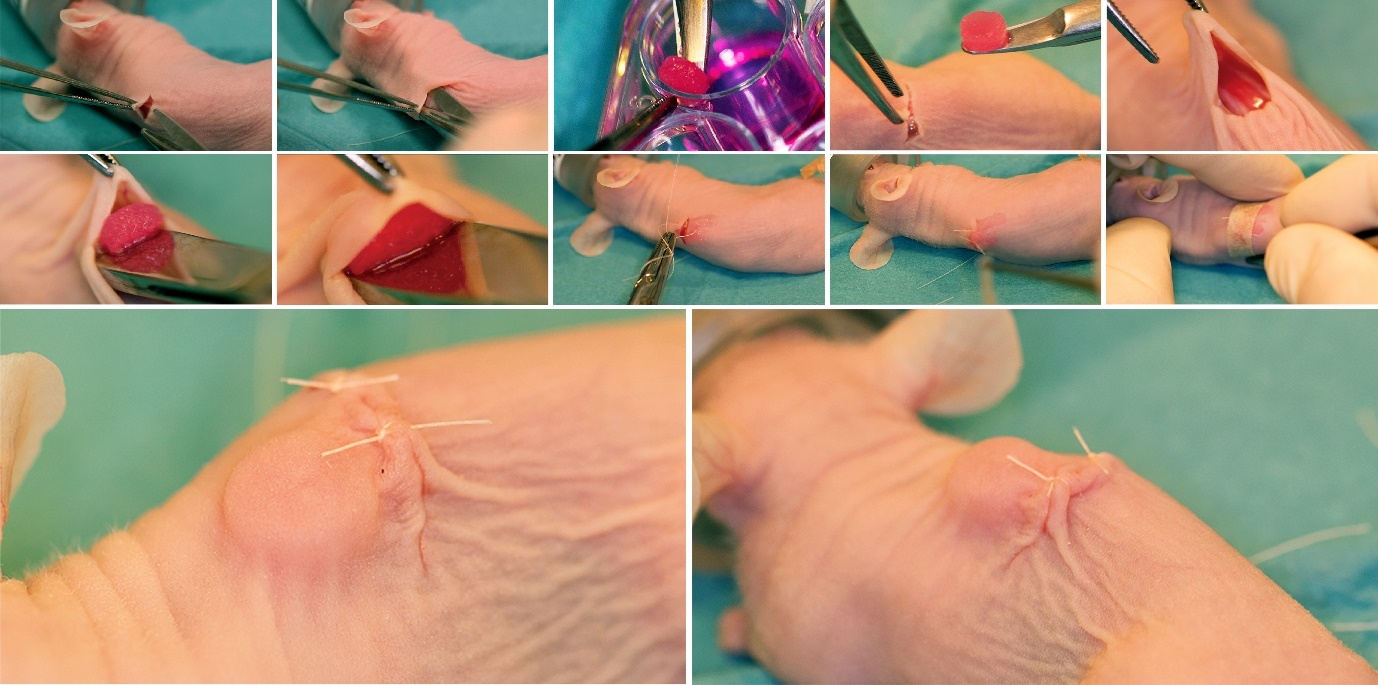


**Figure S10.** In vivo implantation of µInk and µInk + HDF. Constructs were implanted into a subcutaneous wound after dorsal incision. The incision was closed using sutures and sterile wound tape.

**Table S1.** Comprehensive list of primary antibodies used for in vitro and in vivo analysis.

| Protein target | Product number | Dilution | Company | Origin |
| --- | --- | --- | --- | --- |
| Collagen I | Ab6308 | 1:500 | Abcam | Cambridge, UK |
| Collagen III | Ab7778 | 1:500 | Abcam | Cambridge, UK |
| Ki67 | Ab15580 | 1:300 | Abcam | Cambridge, UK |
| Laminin V | Ab78286 | 1:200 | Abcam | Cambridge, UK |
| Pro-Collagen I | MAB1912 | 1:500 | Sigma-Aldrich | Missouri, US |
| α-SMA | A-2547 | 1:400 | Sigma-Aldrich | Missouri, US |
| Collagen IV | Ab6586 | 1:500 | Abcam | Cambridge, UK |
| Fibrillin | MA5-12770 | 1:400 | Thermo Fisher | Massachusetts, US |
| Elastin | Ab2043 | 1:100 | MilliporeSigma | Massachusetts, US |
| HNA | 235-1R | 1:1000 | Thermo Fisher | Massachusetts, US |
| CD31 | 89C2 | 1:1000 | Cell Signaling Technology | Massachusetts, US |
| Phalloidin | A22283 | 1:2000 | Molecular Probes | Oregon, US |

**Table S2.** List of TaqMan assays and their respective target genes.

| Target gene | Assay ID |
| --- | --- |
| HMBS | Hs00609297_m1 |
| ACTA2 | Hs00909449_m1 |
| COL1A1 | Hs00164004_m1 |
| COL3A1 | Hs00943809_m1 |
| COL4A6 | Hs00361494_m1 |
| ELN | Hs01091782_m1 |
| FBN1 | Hs00973198_m1 |
| LAMA3A | Hs00165042_m1 |
| LAMB3 | Hs00165078_m1 |
| LAMC2 | Hs01043711_m1 |
